# Association of Clinician Diagnostic Performance With Machine Learning–Based Decision Support Systems: A Systematic Review

**DOI:** 10.1001/jamanetworkopen.2021.1276

**Published:** 2021-03-11

**Authors:** Baptiste Vasey, Stephan Ursprung, Benjamin Beddoe, Elliott H. Taylor, Neale Marlow, Nicole Bilbro, Peter Watkinson, Peter McCulloch

**Affiliations:** 1 Nuffield Department of Surgical Sciences, University of Oxford, Oxford, United Kingdom; 2 Department of Radiology, University of Cambridge, Cambridge, United Kingdom; 3 Faculty of Medicine, Imperial College London, London, United Kingdom; 4 Oxford University Hospitals NHS Foundation Trust, Oxford, United Kingdom; 5 Department of Surgery, Maimonides Medical Center, Brooklyn, New York; 6 Critical Care Research Group, Nuffield Department of Clinical Neurosciences, University of Oxford, Oxford, United Kingdom

## Abstract

**Importance:**

An increasing number of machine learning (ML)–based clinical decision support systems (CDSSs) are described in the medical literature, but this research focuses almost entirely on comparing CDSS directly with clinicians (human vs computer). Little is known about the outcomes of these systems when used as adjuncts to human decision-making (human vs human with computer).

**Objectives:**

To conduct a systematic review to investigate the association between the interactive use of ML-based diagnostic CDSSs and clinician performance and to examine the extent of the CDSSs’ human factors evaluation.

**Evidence Review:**

A search of MEDLINE, Embase, PsycINFO, and grey literature was conducted for the period between January 1, 2010, and May 31, 2019. Peer-reviewed studies published in English comparing human clinician performance with and without interactive use of an ML-based diagnostic CDSSs were included. All metrics used to assess human performance were considered as outcomes. The risk of bias was assessed using Quality Assessment of Diagnostic Accuracy Studies (QUADAS-2) and Risk of Bias in Non-Randomised Studies–Intervention (ROBINS-I). Narrative summaries were produced for the main outcomes. Given the heterogeneity of medical conditions, outcomes of interest, and evaluation metrics, no meta-analysis was performed.

**Findings:**

A total of 8112 studies were initially retrieved and 5154 abstracts were screened; of these, 37 studies met the inclusion criteria. The median number of participating clinicians was 4 (interquartile range, 3-8). Of the 107 results that reported statistical significance, 54 (50%) were increased by the use of CDSSs, 4 (4%) were decreased, and 49 (46%) showed no change or an unclear change. In the subgroup of studies carried out in representative clinical settings, no association between the use of ML-based diagnostic CDSSs and improved clinician performance could be observed. Interobserver agreement was the commonly reported outcome whose change was the most strongly associated with CDSS use. Four studies (11%) reported on user feedback, and, in all but 1 case, clinicians decided to override at least some of the algorithms’ recommendations. Twenty-eight studies (76%) were rated as having a high risk of bias in at least 1 of the 4 QUADAS-2 core domains, and 6 studies (16%) were considered to be at serious or critical risk of bias using ROBINS-I.

**Conclusions and Relevance:**

This systematic review found only sparse evidence that the use of ML-based CDSSs is associated with improved clinician diagnostic performance. Most studies had a low number of participants, were at high or unclear risk of bias, and showed little or no consideration for human factors. Caution should be exercised when estimating the current potential of ML to improve human diagnostic performance, and more comprehensive evaluation should be conducted before deploying ML-based CDSSs in clinical settings. The results highlight the importance of considering supported human decisions as end points rather than merely the stand-alone CDSSs outputs.

## Key Points

**Question:**

Is clinician diagnostic performance associated with the use of machine learning–based clinical decision support systems?

**Findings:**

In this systematic review of 37 studies, no robust evidence was found to suggest an association between the use of machine learning–based clinical algorithms to support rather than replace human decision-making and improved clinician diagnostic performance.

**Meaning:**

Caution should be observed when estimating the current ability of machine learning algorithms to affect patient care, and emphasis on the evaluation of the human-computer interaction is needed.

## Teaser

This systematic review examines the association between use of machine learning–based clinical decision support systems and clinician performance.

## Introduction

Artificial intelligence has been a popular term in the medical literature and health care industry for some time. Although we are still far from true artificial intelligence, advances in mathematical modeling and computing power have led to an increase in the number of published algorithms. Claims regarding the potential of artificial intelligence in medicine range from being of use to clinicians in their decision-making to artificial intelligence outperforming human experts. Funding for artificial intelligence in health care increases year after year,^1^ and regulatory agencies are approving a growing number of software as medical devices (SaMDs) based on advanced machine learning (ML) algorithms, mainly in medical imaging.^2^ Recent evidence suggests that the best-performing systems are now matching human experts’ performance.^3^ However, few randomized clinical trials or prospective studies have been carried out, and most nonrandomized trials in the field are at high risk of bias.^4^

Machine learning–based clinical decision support systems (CDSSs) are a category of SaMDs designed to support health professionals’ decision-making by providing patient or problem-specific information learned from an ideally large number of clinical cases during a training process. Despite their name, most CDSSs are currently evaluated exclusively against human experts but rarely on their outcome when used with human clinicians of different seniority. Demonstrating that computers can be as good as humans for diagnostic tasks has some useful applications, notably for large population screening in which patients may otherwise not be able to see a physician in a timely manner. Nevertheless, this approach neglects an important factor in any medical encounter: the human clinician. As long as physicians hold the ultimate responsibility for signing off a diagnosis or treatment plan, it will be their interpretation of a CDSS output—not the output itself—that will affect patient care. Human decision-making is known to be influenced by numerous external factors and cognitive bias.^5,6,7^ It would be unwise to assume without further evidence that a human operator would follow a diagnostic CDSS recommendation without question. Extending this argument further, we also have little evidence on how patients would react to fully automated diagnoses or treatment planning. It is therefore important to evaluate any new CDSSs in terms of its performance when used in interactive collaboration with a human clinician and not solely on its performance in silico (ie, on a test data set).

Previous systematic reviews have investigated the association of CDSSs with clinician performance or its surrogate clinical outcomes.^8,9,10,11,12^ However, most of the included studies described systems whose parameters were defined by their developers or diagnosis generators based on handcrafted knowledge bases, hence not fully representing the true promise of ML: to become better than its creator by “learn[ing] without being explicitly programmed.”^13^ In this systematic review, we investigated the current evidence regarding the association between the use of ML-based diagnostic CDSSs and human performance and the ways these systems are evaluated by including all retrieved studies comparing human clinicians performing a diagnostic task with and without ML-based CDSS assistance.

## Methods

### Search Strategy and Selection Criteria

We conducted a systematic review of the literature, and this study followed the relevant sections of the Preferred Reporting Items for Systematic Reviews and Meta-analyses (PRISMA) reporting guideline.^14^ The study is registered with PROSPERO (CRD42019140075).

A search strategy built around 4 additive concepts (machine learning, decision support system, clinician, and performance evaluation) was designed with the support of a specialist librarian and can be found in the study protocol (eAppendix 1 in the Supplement). The search was conducted in MEDLINE, Embase, and PsycINFO for the period between January 1, 2010, and May 31, 2019. The initial search was conducted on May 20, 2019, and the last search to identify possible late indexation within the specified time window was conducted on June 1, 2020. One round of a systematic forward and backward references search was conducted for all included studies. An additional search was performed using the names of algorithms recently approved by the US Food and Drug Administration. A grey literature search including the World Health Organization International Clinical Trials Registry Platform, conference abstracts (from 2017 onward), and the Cochrane Central Register of Controlled Trials was performed using an adapted search strategy (eAppendix 2 in the Supplement).

Inclusion criteria were peer-reviewed articles published in the English language, human physicians as the study population, the interactive use of an ML-based diagnostic CDSSs as intervention, human physicians without CDSSs as a control, any variable used to measure human performance as the main outcome, any variable to measure the stand-alone computer performance (ie, the performance achieved by the computer outputs without subsequent human intervention), and any variable describing the evaluation of the CDSS by the human operator as a secondary outcome. A CDSS was considered diagnostic if its output produced qualitative information (eg, benign vs malignant) about the nature of a lesion or if the detection of a lesion was in itself sufficient to pose a diagnosis and influence a therapeutic choice (eg, the presence of pulmonary emboli). Exclusion criteria were monitoring, alert, or detection-only systems; systems based on validated scores only; systems based on natural language processing only; and systems relying on handcrafted knowledge or rule bases. The specific definition of key concepts and complete exclusion criteria can be found in eAppendix 1 in the Supplement. All retrieved titles and abstracts were independently screened by at least 2 of us (B.V., S.U., E.H.T., N.M., and N.B.). Conflicts were adjudicated by a third reviewer (S.U. or B.B.). Full-text articles were independently reviewed for eligibility by at least 2 of us (B.V., S.U., B.B., E.H.T., N.M., and N.B.). Conflicts were resolved in consensus. The abstract screening and full-text review were conducted using the Covidence software.^15^

Data were extracted about the study population, patient population, data set characteristics, experiment description, system characteristics, metrics assessing human performance, metrics assessing computer performance, and study funding. The full list of data items can be found in eAppendix 1 in the Supplement. Investigators were not contacted. The risk of bias for each included study was assessed using the Quality Assessment of Diagnostic Accuracy Studies (QUADAS-2) tool as modified by Riches^8,16^ and the Risk of Bias in Non-Randomised Studies–Intervention (ROBINS-I) tool.^17^ QUADAS-2 was used to assess the risk of bias regarding the claims of CDSS diagnostic accuracy, and ROBINS-I was used to consider the risk of bias in the results assessing difference in performance. Studies were included in the analysis independently of their risk of bias. Data extraction and bias assessments were all conducted independently by at least 2 of us (B.V., S.U., B.B., E.H.T., N.M., and N.B.) using piloted forms. Conflicts were resolved by consensus. To ensure consistency, the main reviewer (B.V.) screened all abstracts and full texts for eligibility, extracted data, and assessed risk of bias on all included studies.

Meta-bias was investigated by searching the World Health Organization International Clinical Trials Registry Platform and Cochrane Central Register of Controlled Trials registers looking for unpublished trials and evidence of selective reporting. The origin of study funding and the presence of a protocol were also considered.

### Data Analysis

Narrative summaries were produced for the primary and secondary outcomes. As per protocol, subgroup analyses were performed for clinicians’ experience level (experienced vs novice), the mathematical model used, the models’ degree of support (single output vs information about process), and the reader paradigm (first vs second reader). First reader support displays the model output at the same time as the clinical data, and second reader support displays the model output after the observer had a chance to make their own decision on a case. An additional subgroup analysis for studies evaluating ML-based CDSSs in a representative clinical environment (clearly reported consecutive or nonaugmented random patient sample and access to the usually available clinical data at the time of decision-making) was performed. Patient-level results were prioritized over lesion-level results for the summary of main results. Patient or lesion types subgroup analyses were summarized separately. Given the heterogeneity of medical conditions, outcomes of interest, and evaluation metrics, no meta-analysis was performed. All studies were included in the analysis irrespective of their risk of bias.

## Results

**Figure 1.**
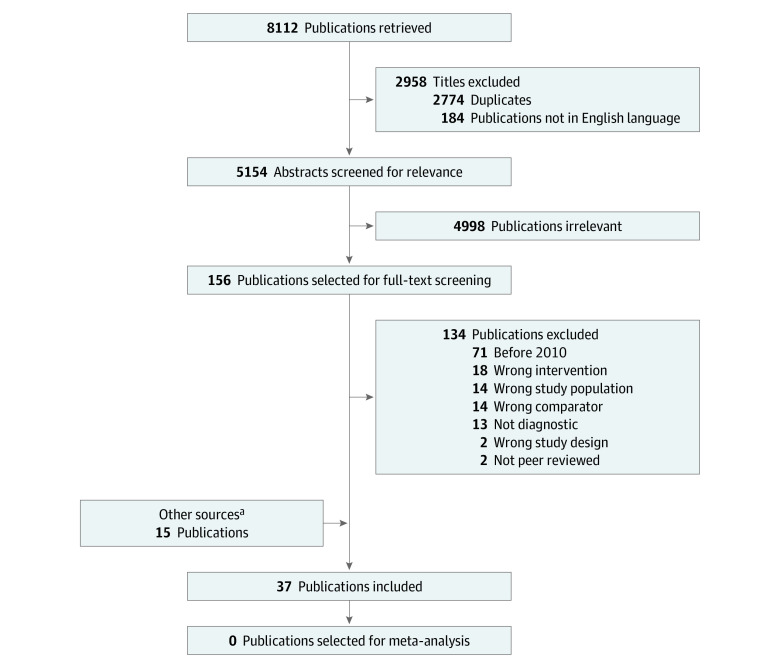
Flowchart of Study Inclusion ^a^Other sources included forward/backward literature search, reference search from relevant literature, trade name search, and conference abstracts or entries in the Cochrane Central Register of Controlled Trials that led to publications.

A total of 8112 titles were identified, of which 2774 were duplicates and 184 were not published in English; 5154 abstracts were screened, and 156 of these were selected for full-text review. Of the 156 studies assessed, 22 were eligible for inclusion. Fifteen additional publications meeting the inclusion criteria were retrieved from other sources, including forward/backward references search, trade names search, related literature references search, and publications tracing from the grey literature search. Thirty-seven publications were eventually included in this review.^18,19,20,21,22,23,24,25,26,27,28,29,30,31,32,33,34,35,36,37,38,39,40,41,42,43,44,45,46,47,48,49,50,51,52,53,54^ Figure 1 presents the PRISMA flowchart.

All included studies described CDSSs based on imaging modalities, with breast and pulmonary diseases being the most common medical conditions. Twenty studies (54%) investigated CDSSs technology with a designated trade name at the time of publication.

Thirty-one studies (84%) assessed a CDSS belonging to the International Medical Device Regulators Forum’s risk category 4 (the highest category),^55^ 25 studies (68%) used a second reader paradigm (see Data Analysis section) for the CDSS support, 8 (22%) used a first reader paradigm, 1 study (3%) used both, and 3 studies (8%) did not specify. Three studies (8%) used the same cases as training and test sets, and 3 studies (8%) did not report clearly on test set independence. The median proportion of events (ie, target condition) in the test set was 44% (interquartile range, 32%-54%). The median number of clinician participants in the observer test was 4 (interquartile range, 3-8), with each reading a median of 123 different cases (interquartile range, 79-300). Table 1 gives an overview of the included studies’ characteristics.

**Table 1.** Characteristics of Included Studies

Source	Medical condition	Algorithm used	IMDRF risk category	No. of sites	Test set sample size^a^	Test set event cases^b^	No. of study participants	Cases read/participant^c^	Reader paradigm	Private sector funding
Aissa et al,^18^ 2018	Melanoma	ClearRead CT (CNN)^d^	4	1	46	46	3	46	First	NA
Aslantas et al,^19^ 2016	Bone metastasis	Perceptron-based ANN	4	2	130	100	1	130	NA	No
Bargalló et al,^20^ 2014	Breast cancer	SecondLook^d^	4	NA	21 321	130	4	8100^e^	Second	NA
Barinov et al,^21^ 2019	Breast cancer	cCAD (ANN)^d^	4	Multiple	500	150	3	450 and 500	First and second	NA
Bartolotta et al,^22^ 2018	Breast cancer	S-Detect (CNN)^d^	4	NA	300	122	4	300	Second	NA
Bien et al,^23^ 2018	Knee musculoskeletal injury	CNN	3	2	120	99	9	120	First	NA
van den Biggelaar et al,^24^ 2010	Breast cancer	SecondLook^d^	3	1	1048	50	2	524^e^	First	No
Blackmon et al,^25^ 2011	Pulmonary embolism	VA10 PE (SVM)	4	NA	79	32	2	79	Second	NA
Cha et al,^26^ 2019	Bladder cancer	CNN^d^	4	NA	123	40	12	123	Second	No
Chabi et al,^27^ 2012	Breast cancer	B-CAD v.2^d^	4	1	160	77	4	160	Second	NA
Cho et al,^28^ 2018	Breast cancer	S-Detect (CNN)^d^	4	1	119	54	2	119	Second	Yes
Choi JH et al,^29^ 2018	Breast cancer	S-Detect (CNN)^d^	4	1	200	12	4	100^e^	Second	No
Choi JS et al,^30^ 2019	Breast cancer	S-Detect (CNN)^d^	4	1	253	80	4	253	Second	No
Cole et al,^31^ 2014	Breast cancer	ImageChecker v.1.0 (CNN) and SecondLook v.1.4^d^	4	Multiple	300 and 300	150 and 150	15 and 14	300 and 300	Second	No
Endo et al,^32^ 2012	Pulmonary nodule	Euclidian distance clustering	4	1	30	23	3	30	NA	NA
Engelke et al,^33^ 2010	Pulmonary embolism	PE-CAD (MIC)^d^	4	NA	58	58	4	58	Second	NA
Giannini et al,^34^ 2017	Prostate cancer	SVM	4	NA	89	35	3	89	First	No
Hwang et al,^35^ 2019	Thoracic pathology	CNN	4	1	200	103	15	200	Second	No
Lindsey et al,^36^ 2018	Wrist fracture	CNN	3	1	300	NA	24	300	Second	Yes
Park et al,^37^ 2019	Breast cancer	S-Detect (CNN)^d^	4	1	100	41	5	100	Second	No
Rodríguez-Ruiz et al,^38^ 2019	Breast cancer	Transpara v.1.3.0 (CNN)^d^	4	2	240	100	14	240	NA	Yes
Romero et al,^39^ 2011	Breast cancer	Image Checker v.5.4 (CNN)^d^	4	1	9389	124	2	4695^e^	Second	NA
Samulski et al,^40^ 2010	Breast cancer	Image Checker v.8.0 (CNN)^d^	4	NA	120	40	9	120	Second	No
Sanchez Gómez et al,^41^ 2011	Breast cancer	SecondLook v.1.1^d^	4	NA	21 855	94	6	3643^e^	Second	No
Sayres et al,^42^ 2019	Diabetic retinopathy	CNN	3	Multiple	1796	213	10	1796	First	Yes
Shimauchi et al,^43^ 2011	Breast cancer	Bayesian ANN	4	2	60	30	6	60	Second	NA
Sohns et al,^44^ 2010	Breast cancer	Image Checker v.2.3 (CNN)^d^	4	NA	303	98	2	303	First	NA
Steiner et al,^45^ 2018	Breast cancer	CNN	4	2	70	38	6	70	First	Yes
Stoffel et al,^46^ 2018	Breast cancer	ViDi Suite v.2.0 (ANN)^d^	4	1	33	11	4	33	First	No
Sun et al,^47^ 2014	Atrial thrombus	ANN	3	1	130	31	5	130	Second	No
Sunwoo et al,^48^ 2017	Brain metastasis	k-means clustering + ANN	4	1	60	30	4	60	Second	No
Tang et al,^49^ 2011	Ischemic stroke	ANN	4	Multiple	71	40	6	40	Second	No
Taylor et al,^50^ 2018	Parkinsonian syndromes	SVM	3	1 and Multiple	55 and 100	33 and 60	2	55 and 100	Second	No
Vassallo et al,^51^ 2019	Lung metastasis	SVM	4	1	225	75	3	225	Second	No
Watanabe et al,^52^ 2019	Breast cancer	cmAssist (CNN)^d^	4	1	122	90	7	120	Second	Yes
Way et al,^53^ 2010	Lung cancers	Linear discriminant analysis	4	1	256	124	6	NA	Second	No
Zhang et al,^54^ 2016	Lymph node cancers	SVM	4	1	178	87	10	178	Second	No

Abbreviations: ANN, artificial neural network; CNN, convolutional neural network; IMDRF, International Medical Device Regulators Forum; MIC, multiple instance classifier; NA, not available; SVM, support vector machine.

^a^ For the observer test with support for clinical decision support systems.

^b^ One case can display multiple events.

^c^ Each case was either seen once or multiple times (with or without assistance) depending on the study.

^d^ Commercial name of proprietary algorithm.

^e^ Mean value.

The 10 most common metrics used to quantify human performance in the included studies were sensitivity (81%), specificity (70%), area under the receiver operating curves (51%), accuracy (38%), interobserver agreement (30%), positive predictive value (PPV) (30%), negative predictive value (30%), reading time (22%), rate of recall for further investigation (11%), and the positive value of further investigations (8%). Equivalent metrics have been aggregated. A full list of evaluation metrics with their occurrence can be found in eTable 1 in the Supplement.

Table 2 reports a summary of the association between CDSS use and the 10 most common human performance evaluation metrics. Three studies reported on more than 1 CDSS (or using the same CDSS in different modalities).^21,31,42^ A total of 107 main results were reported with statistical significance, and 41 were reported without it. Most studies defined statistical significance at *P* < .05, with some applying correction for multiple comparisons. Of the results reported with statistical significance, 54 studies (50%) showed an increase in their metrics, 4 (4%) reported a decrease, and 49 (46%) noted no change or an unclear change. The area under the receiver operating curves, accuracy, interobserver agreement, and PPV were usually increased with interobserver agreement showing the clearest change. The sensitivity, specificity, negative predictive value, rate of recall for further investigation, and PPV of further investigations remained unchanged in most cases, and the CDSS association with reading time showed no clear pattern. Sixteen studies also reported analyses on subgroups of patients or lesion types. A summary of these additional analyses can be found in eTable 2 in the Supplement, and a detailed list of the included studies’ results are reported in eTable 3 in the Supplement.

**Table 2.** Association Between ML-Based CDSS Use and Clinician Performance^a^

Metric category	Results reported with statistical significance, No.			Results reported without statistical significance, No.			Total CDSSs evaluated, No.^b^
	Increase overall or for ≥50% of the participants	No change or unclear change for the group	Decrease overall or for ≥50% of the participants	Increase overall or for ≥50% of the participants	No change or unclear change for the group	Decrease overall or for ≥50% of the participants	
Sensitivity	10	11	1	9	1	0	32
Specificity	6	11	1	3	2	5	28
Area under the receiver operating curves	13	7	0	1	0	0	21
Accuracy	8	4	0	5	1	0	18
Interobserver agreement	7	2	0	2	0	0	11
Positive predictive value	5	3	0	2	0	2	12
Negative predictive value	3	5	0	3	1	0	12
Reading time	2	2	2	0	1	1	8
Recall for further investigations	0	2	0	1	0	0	3
Positive predictive value of further investigations	0	2	0	0	0	1	3

Abbreviations: CDSSs, clinical decision support systems; ML, machine learning.

^a^ Number of main results reported for the 10 most commonly used metrics groups comparing computer-assisted clinicians with clinicians alone.

^b^ Three studies reported on more than 1 CDSS or used the same CDSS in different modalities.

In the 6 studies^20,22,24,29,39,41^ evaluating CDSSs in a representative clinical environment for the same 10 evaluation metrics, 20 results were reported with statistical significance. Of these, 16 (80%) showed no difference in performance, and 4 (20%) reported an increase in sensitivity, area under the receiver operating curves, PPV, or interobserver agreement (eTable 4 in the Supplement).

In 19 studies in which a comparison was possible, CDSSs were more often associated with an increase in performance for less experienced clinicians compared with their senior colleagues (eTable 5 in the Supplement). The reader paradigm also appeared to be associated with human performance, with studies investigating CDSSs in the second reader mode appearing to be more often associated with an increase in the metrics (eTable 6 in the Supplement). The subgroup analyses according to the mathematical model used (eTable 7 in the Supplement) and degree of support (eTable 8 in the Supplement) produced no additional findings.

Twenty-seven studies reported on CDSS stand-alone performance. With the exception of 1 unclear case,^50^ human participants always decided to override at least some of the CDSS recommendations. Of the 75 main results reported using the 10 most commonly applied metrics, the human contribution changed the system performance in 70 cases (93%). Compared with the stand-alone computer performance, adding human intelligence increased the metrics value in 45 cases (60%) and decreased it in 25 cases (33%). Only 3 results (4%) mentioned statistical significance; of these, 1 showed no statistical difference and 2 noted a significant increase in accuracy. An overview of these results is reported in Table 3, a summary of the subgroup analyses in eTable 9 in the Supplement, and a detailed list of the results in eTable 10 in the Supplement.

**Table 3.** Association Between Human Contribution and System Performance^a^

Metric category	Results reported with statistical significance, No.			Results reported without statistical significance, No.			Total CDSSs evaluated, No.^b^
	Increase overall or for ≥50% of the participants	No change or unclear change for the group	Decrease overall or for ≥50% of the participants	Increase overall or for ≥50% of the participants	No change or unclear change for the group	Decrease overall or for ≥50% of the participants	
Sensitivity	0	0	0	11	2	10	23
Specificity	0	0	0	11	2	5	18
Area under the receiver operating curves	0	1	0	5	0	7	13
Accuracy	2	0	0	6	0	1	9
Interobserver agreement	0	0	0	6	0	0	6
Positive predictive value	0	0	0	0	0	0	0
Negative predictive value	0	0	0	4	0	2	6
Reading time	0	0	0	0	0	0	0
Recall for further investigations	0	0	0	0	0	0	0
Positive predictive value of further investigations	0	0	0	0	0	0	0

Abbreviation: CDSSs, clinical decision support systems.

^a^ Number of main results reported for the 10 most commonly used metrics groups comparing computer-assisted clinicians with stand-alone computers.

^b^ Three studies reported on more than 1 CDSS or used the same CDSS in different modalities.

Of the 37 included studies, 15 (41%) attempted to increase the interpretability of the model by presenting some of the intermediary calculations leading to the models’ final output, and 13 studies (35%) included users’ training before starting data collection. Four (11%) reported on user feedback about the CDSSs, of which 3 (8%) gathered feedback through a formalized process. Van den Biggelaar et al^24^^(p501)^ asked study participants to indicate on their case evaluation forms if the CDSS marks “added valuable diagnostic information to their own original evaluation” but did not report on this outcome. Taylor et al^50^^(p5)^ designed open and closed question interviews to “provide an insight into the CADx [computer-aided diagnosis]-radiologist relationship [and] to assess the effects of the CADx software on clinician decision-making.” The study participants reported good agreement between their decision and the CDSS outputs, with a small to moderate influence on their reporting decision. The participants also considered it would be of small to moderate benefit if the CDSS would display more information on how it generates its decision and thought CDSSs could be of moderate to substantial benefit to support training and improve inexperienced clinicians’ performance. Endo et al^32^ invited study participants to give direct feedback on CDSS outputs by grading their relevance in the context of a specific task; 87% of the outputs were judged satisfactory. Additional human factor–related characteristics of the included studies can be found in eTable 11 in the Supplement.

**Figure 2.**
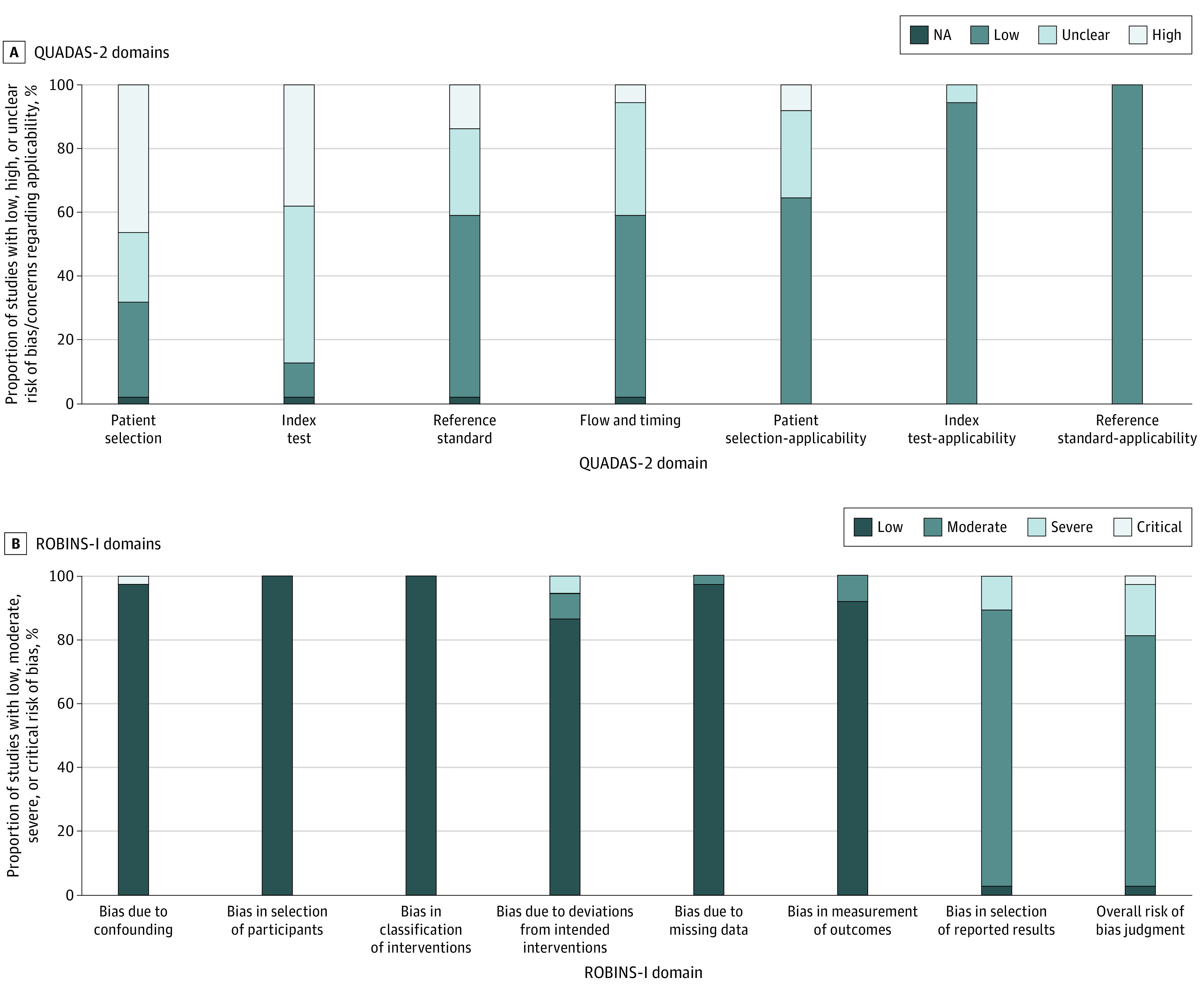
Distribution of the Risk of Bias Scores The total of 100% represents 37 included studies in the Quality Assessment of Diagnostic Accuracy Studies (QUADAS-2) (A) and Risk of Bias in Non-Randomised Studies–Intervention (ROBINS-I) (B) domains.

Using QUADAS-2, 28 studies (76%) were rated as having high risk of bias in at least 1 of the 4 core domains, and none were considered to have a low risk of bias in all 4 core domains. Patient selection and the index test were the 2 domains most frequently found at high risk of bias. Using ROBINS-I, 6 studies (16%) were rated as having serious or critical risk of bias due to cofounding, deviation from the intended interventions, or likely selection of the reported results. Only 1 study was considered to be at low risk of bias in all 7 domains.^47^ Figure 2 shows the overall risk of bias assessment for each category of these tools.

Six studies (16%) reported private sector funding, and 12 (32%) gave no or unclear information about their source of funding. Only 2 studies (5%) referenced a study protocol.^47,54^ The grey literature search retrieved 1 randomized clinical trial protocol (with expected completion after the present review’s search period), 1 conference abstract (leading to a publication after the present review’s search period),^56^ and 1 conference abstract that did not lead to any publication.

## Discussion

This systematic review found no robust evidence that the use of ML-based algorithms was associated with better clinician diagnostic performance. The evidence for any conclusion was weak because of a high risk of bias in many of the studies and a low number of study participants. Almost half of all results reported with statistical significance showed no significant difference in performance with or without the use of CDSSs. In studies conducted in a clearly reported representative clinical environment, this observation was even clearer, with 80% of the designated results showing no statistically significant change in performance. These findings corroborate the conclusions of several other studies assessing the outcome of CDSS use in mammogram screening across large populations, in which few or no benefits were found.^57,58,59^ In a cross-specialty review like ours, expressing a straightforward judgment about the benefits of a CDSS is often difficult as it heavily depends on factors such as common clinical practice in a field or the prevalence of the target condition. This factor is the reason why we summarized the association between the use of CDSSs and clinician performance by metrics, as they enable readers to decide whether specific changes are desirable in their specialty. The interobserver agreement was the metric whose change appeared to be the most clearly associated with the use of CDSSs. The use of CDSSs also appeared to have a more marked association with increased performance for less experienced clinicians and to be associated with increased interobserver agreement between clinicians of different experience levels. In this way, CDSSs need not be solely used to outperform the most experienced clinicians but could be targeted by design toward those with less experience who may receive more benefit.

Little consideration was given to human factors in included studies. This outcome is surprising as human clinicians should be the main beneficiaries of the systems tested. In only 13 studies were the observers trained on the CDSS before the test. Given the likely existence of a learning (or trust) curve as observed by Rodríguez-Ruiz et al,^38^ this omission might well have distorted some of the results. User feedback was reported in only 4 studies, hence hindering any iterative improvement in the human-computer interaction. This outcome is in contrast to other safety-critical industries, such as the aviation or energy sectors, where human factors principles have been commonly used for years.^60,61,62,63,64^

In all but 1 study in which the information was available, human operators decided to override at least some of the system recommendations, and it remains unclear to what extent human intelligence influences the overall system performance. These 2 observations highlight that computer simulations alone are insufficient to define the effectiveness and safety profile of a CDSS. In clinical situations in which humans have the responsibility for a diagnostic or therapeutic choice, they will, consciously or not, factor in other variables than the CDSS outputs and possibly prioritize their own clinical judgment in case of conflict. Therefore, it is the human processing of algorithm outputs, rather than the outputs themselves, that will affect patient care. Thus, it is important to evaluate this shared decision-making process rather than the CDSS stand-alone performance.

Many of the included studies were at high risk of bias, echoing the results of a recent review assessing studies comparing deep learning–based algorithms to clinicians.^4^ This elevated risk of bias was mainly attributed to 3 factors: (1) the lack of prospectively or randomly selected case samples, (2) the absence during the test of clinical data otherwise available in real-life settings, and (3) the absence of a protocol. Moreover, the generalizability of the studies’ findings was undermined by the absence of any power calculation and the median number of participants being only 4. In many cases, we also observed confusion between statistical significance at the patient and practitioner levels. Bootstrapping the clinical cases to produce a *P* value would not, for example, give any indication about the generalizability of findings to other clinicians. Instead, it would assess the likelihood that the same clinicians would display similar improvement with a new sample of patients.

In addition to the issues already outlined, there was a marked heterogeneity in the metrics used to assess CDSSs. Together, these inconsistencies make a reliable comparison of the different systems almost impossible. The issue of performance comparability is well known to the field and initiated the creation of data challenges, notably in medical image analysis, to evaluate how competing algorithms perform on common data sets.^65,66^ This harmonization work should now be extended to the next phases of CDSS evaluation pathways, particularly when first used with human clinicians. Reporting guidelines would offer a practicable solution to this end.

### Strengths and Limitations

The methodologic approach followed best practice standards for systematic reviews, and each step of the process was performed independently by at least 2 reviewers. This study is, to our knowledge, the first to put human clinicians, rather than the algorithms, at the forefront of a systematic review about the clinical use of ML-based CDSSs. This approach provides important information that nuances a commonly portrayed view that artificial intelligence may soon substantially improve clinician diagnostic performance across specialties. This approach also highlights the current lack of consideration of human factors when assessing the potential benefits of new CDSSs. In addition, this review provides material that can inform the development of further guidance on ML-based CDSS evaluation, complementing existing or upcoming reporting guidelines.^67,68,69,70^ Such guidance will be particularly relevant for safety and effectiveness evaluations before the execution of large-scale clinical trials.

This review has limitations. It is possible that some relevant literature was not retrieved owing to (1) the heterogeneous description of the target CDSSs across medical specialties, (2) the use of commercial names only in many studies, and (3) the only recent categorization of this technology in specialized search engines (*machine learning* was added as a MeSH term in PubMed in 2016). We addressed these issues by conducting a forward and backward literature search of the included studies as well as an additional search for common or new commercial names. Given the broad range of CDSSs evaluated herein, certain inclusion criteria had to be defined very precisely, and some of these definitions are debatable because there is no broad consensus in the literature.

## Conclusions

This systematic review of the literature provides findings to inform current and future debate about the evaluation of ML in health care. We found no robust evidence to suggest that the use of ML-based CDSSs is associated with improved diagnostic performance among clinicians in representative clinical environments. We also highlighted that most of the studies on this topic were at high or unclear risk of bias and had a low number of participants. In addition, we observed that the human operators almost always decided to override at least some of the CDSS recommendations. Therefore, we recommend more thorough evaluation of ML-based CDSSs and that more consideration be given to the human component of assisted diagnosis. These changes in practice should be guided by accepted principles of trial conduct and reporting to avoid repetition of errors noted in the current literature. Increased regulatory scrutiny also has an important role in ensuring a safe and efficient translation to the patient bedside. The results of this review should not be interpreted as tarnishing the prospects of ML-based diagnostic CDSSs. Rather, we encourage qualitative improvements in future research. Better methodologies and evaluations would allow CDSSs to showcase their full potential and ultimately improve patient care.

## Notes

Supplement. **eAppendix 1.** Systematic Review Protocol **eAppendix 2.** Modified Search Strategies **eTable 1.** Metrics Used to Evaluate the Impact of ML-Based CDSS on Human Performance **eTable 2.** Impact of ML-Based CDSS on Clinician Performance in Patients or Lesions Subgroup **eTable 3.** Complete List of the Included Studies’ Results for the Primary Outcome **eTable 4.** Impact on Clinician Performance of the Six ML-Based CDSS Evaluated in Representative Clinical Environment **eTable 5.** Association Between Clinicians’ Level of Experience and Performance Changes When Using ML-Based CDSS **eTable 6.** Impact on Clinician Performance of ML-Based CDSS According to the Reader Paradigm (First Reader/Second Reader) **eTable 7.** Impact on Clinician Performance of ML-Based CDSS According to the Mathematical Model Used (Neural Networks/Other Models) **eTable 8.** Impact on Clinician Performance of ML-Based CDSS According to the Outputs’ Level of Support (Single Output/Explanatory Output) **eTable 9.** Impact of the Human Contribution on the System Performance in Patients or Lesions Subgroups **eTable 10.** Complete List of the Included Studies’ Results for the Secondary Outcome (Assisted Human Performance vs Stand-Alone Computer Performance) **eTable 11.** Characteristics Relevant to the Human Factors Evaluation of the Included Studies
