# Supplementary material for: Association of Clinician Diagnostic Performance With Machine Learning–Based Decision Support Systems: A Systematic Review
Source: JAMA Netw Open. 2021 Mar 11;4(3):e211276. doi: 10.1001/jamanetworkopen.2021.1276 (PMC7953308; doi:10.1001/jamanetworkopen.2021.1276)
Supplement: Supplement. — eAppendix 1. Systematic Review Protocol eAppendix 2. Modified Search Strategies eTable 1. Metrics Used to Evaluate the Impact of ML-Based CDSS on Human Performance eTable 2. Impact of ML-Based CDSS on Clinician Performance in Patients or Lesions Subgroup eTable 3. Complete List of the Included Studies’ Results for the Primary Outcome eTable 4. Impact on Clinician Performance of the Six ML-Based CDSS Evaluated in Representative Clinical Environment eTable 5. Association Between Clinicians’ Level of Experience and Performance Changes When Using ML-Based CDSS eTable 6. Impact on Clinician Performance of ML-Based CDSS According to the Reader Paradigm (First Reader/Second Reader) eTable 7. Impact on Clinician Performance of ML-Based CDSS According to the Mathematical Model Used (Neural Networks/Other Models) eTable 8. Impact on Clinician Performance of ML-Based CDSS According to the Outputs’ Level of Support (Single Output/Explanatory Output) eTable 9. Impact of the Human Contribution on the System Performance in Patients or Lesions Subgroups eTable 10. Complete List of the Included Studies’ Results for the Secondary Outcome (Assisted Human Performance vs Stand-Alone Computer Performance) eTable 11. Characteristics Relevant to the Human Factors Evaluation of the Included Studies [file jamanetwopen-e211276-s001.pdf]

Supplementary Online Content

Vasey B, Ursprung S, Beddoe B, et al. Association of clinician diagnostic performance with machine learning–based decision support systems: a systematic review. *JAMA Netw Open*. 2021;4(3):e211276. doi:10.1001/jamanetworkopen.2021.1276

eAppendix 1. Systematic Review Protocol

eAppendix 2. Modified Search Strategies

eTable 1. Metrics Used to Evaluate the Impact of ML-Based CDSS on Human Performance

eTable 2. Impact of ML-Based CDSS on Clinician Performance in Patients or Lesions Subgroup

eTable 3. Complete List of the Included Studies' Results for the Primary Outcome

eTable 4. Impact on Clinician Performance of the Six ML-Based CDSS Evaluated in Representative Clinical Environment

eTable 5. Association Between Clinicians' Level of Experience and Performance Changes When Using ML-Based CDSS

eTable 6. Impact on Clinician Performance of ML-Based CDSS According to the Reader Paradigm (First Reader/Second Reader)

eTable 7. Impact on Clinician Performance of ML-Based CDSS According to the Mathematical Model Used (Neural Networks/Other Models)

eTable 8. Impact on Clinician Performance of ML-Based CDSS According to the Outputs' Level of Support (Single Output/Explanatory Output)

eTable 9. Impact of the Human Contribution on the System Performance in Patients or Lesions Subgroups

eTable 10. Complete List of the Included Studies' Results for the Secondary Outcome (Assisted Human Performance vs Stand-Alone Computer Performance)

eTable 11. Characteristics Relevant to the Human Factors Evaluation of the Included Studies

This supplementary material has been provided by the authors to give readers additional information about their work.

© 2021 Vasey B et al. *JAMA Network Open.*

eAppendix 1. Systematic review protocol

This is the fifth version of the protocol, last modified on the 16.03.20 (original: 24.06.19).
This protocol follows the recommendations of the PRISMA-P 2015 statement.[1,2]

ADMINISTRATIVE INFORMATION

Title

Effects of Clinical Diagnostic Decision Support Systems based on Machine Learning on Physicians' Performance – Protocol for a Systematic Review

Registration

This protocol for a systematic review is registered with the International Prospective Register of Systematic Reviews (PROSPERO). The registration was made on the 24th of June 2019 and not updated since. The registration number is 140075.

Authors

First reviewer: **Baptiste Vasey**, Nuffield Department of Surgical Sciences, University of Oxford, Oxford, UK
baptiste.vasey@nds.ox.ac.uk

Second reviewers: **Nicole Bilbro**, Maimonides Medical Center, Brooklyn, NY, USA
nicole.bilbro@maimonidesmed.org

Neale Marlow, Nuffield Department of Surgical Sciences, University of Oxford, Oxford, UK
neale.marlow@trinity.ox.ac.uk

Stephan Ursprung, Department of Radiology, University of Cambridge, Cambridge UK
su263@cam.ac.uk

Benjamin Beddoe, Faculty of Medicine, Imperial College, London, UK
benjamin.beddoe15@imperial.ac.uk

Elliott Taylor, Nuffield Department of Surgical Sciences, University of Oxford, Oxford, UK
elliott.taylor@trinity.ox.ac.uk

Guarantor: **Prof Peter McCulloch**, Nuffield Department of Surgical Sciences, University of Oxford, Oxford, UK
peter.mcculloch@nds.ox.ac.uk

Corresponding author: **Baptiste Vasey**
Nuffield Department of Surgical Sciences
University of Oxford
Level 5, Room 5402
John Radcliffe Hospital
Headington
Oxford, OX3 9DU

© 2021 Vasey B et al. *JAMA Network Open.*

Contributions

BV designed the search strategy, wrote the present protocol and will be first reviewer during the abstracts screening and full texts review phases. NB supported the development of the search strategy, reviewed the protocol and will be second reviewer during the abstracts screening and full texts review phases. NM reviewed the protocol and will be second reviewer during the abstracts screening and full texts review phases. SU reviewed the protocol and will be second reviewer and resolve conflicts during the abstracts screening and full texts review phases. BB and NM will be second reviewers. PM reviewed the protocol, will resolve conflicts during the abstracts screening and full texts review phases and is the guarantor. All authors will contribute to the data extraction and analysis, and to the writing of the final manuscript.

Support

Outreach Librarian — **Tatjana Petrinic** (Bodleian Libraries, University of Oxford) supported the development of the search strategy and advised on the systematic review methodology

Funding — No specific funding was provided for this systematic review.

Amendments

All amendments to the present protocol shall be documented under this section and in the PROSPERO record. All amendments shall by complemented by a description, a rational and a date for the change.

13.07.19 Following a request from the PROSPERO administrator, the synthesis plan in the "Data synthesis" section was described in more details.

Old: "Due to the expected heterogeneity of the systematic review's target studies, the authors do not plan a meta-analysis at the time of writing this protocol. A descriptive synthesis and an analysis of the reported outcomes in line with the systematic review's objectives will be performed. Subgroups analysis will be performed according to algorithm design, degree of support, medical specialty and any other coherent groups that would emerge from the included studies."

New: "A narrative synthesis of the reported outcomes in line with the systematic review's objectives will be performed, including differences in performance between the intervention and control groups as well as between the intervention group and the computer system alone. Underlying factors possibly explaining changes in effect size or direction will be investigated. The authors expect a noticeable variability in the metrics used to assess performance. A summary table of the these metrics will be presented. Qualitative data will be presented descriptively as recommended in the PRISMA elaboration and explanation document."
"If a subgroup is sufficiently homogenous in term of study population and performance metrics, a quantitative synthesis of the performance metrics will be considered. The minimal number of studies required for this synthesis will depend on the number of participants in each study."

09.10.19 The intervention criteria have been clarified to address uncertainties arisen during abstracts screening.

Old: "Interactive use of a decision support system based on clinical data and machine learning algorithms to improve diagnosis or diagnostic investigations planning. In the context of this review, machine learning algorithms are defined as algorithms that have the ability to independently learn from clinical data knowledge unknown to their programmers and to generate outputs that have not been explicitly programmed."

New: "Interactive use of a decision support system based on clinical data and machine learning algorithms to improve diagnosis or diagnostic investigations planning. In the context of this review, machine learning algorithms are defined as algorithms that have the ability to independently learn, from clinical data, knowledge unknown to their programmers and to generate outputs that have not been explicitly programmed. Machine learning models considered as general medical statistics, such as linear regression and logistic regression are not included. Diagnosis is defined as "the identification of the nature of an illness" (Oxford Dictionary)."

09.10.19: Two new second reviewers are added.

Benjamin Beddoe, Elliott Taylor

26.11.19: The list of data items to be extracted is modified to reflect the feedback generated during the piloting of the extraction table.

Old: “The following data will be extracted if present:
- study population: number, specialty, seniority
- patient population: in-/outpatient, type of medical conditions, centre size
- dataset: type of sample, sample size and number of events (for training and validation sets), source
- experiment: number of cases per physician, chronology, blinding process, familiarity with the system
- main purpose of the decision support system
- system characteristics: degree of support (tailored information display, highlighted information display, choice of several recommendations, unique recommendation; this scale will be adapted to better reflect the variety of decision support systems encountered), type of recommendations, timing of the recommendation, mathematical model used, attempts to increase the interpretability of the model
- metrics of human performance: type and value of all the metrics used to assess human performance with and without the support system, including, but not limited to, sensitivity, specificity, area under the receiver operating characteristic curve (AUC), positive and negative predictive values, precision, accuracy, recall, position, time to decision, inter- and intra-operator variability, usability, clinical outcomes (mortality, morbidity, adverse events) and institutional outputs (average cost of treatment, length of stay)
- metrics of computer performance: type and value of all the metrics used to assess the performance of the decision support system alone, including, but not limited to, sensitivity, specificity, AUC, positive and negative predictive values, precision, accuracy, recall, position and time to decision.
- study funding: provenance, amount
- existence of a published study protocol”

New: “The following data will be extracted if present:
- study population: number, specialty, seniority
- patient population: type of medical conditions, number of different hospital sites
- dataset: type of sample, sample size and number of events (for training and validation sets), independence of training and test sets
- experiment: task to be performed, experimental design, number of cases per physician, timing of support, gold standard comparison, familiarity with the system.
- main purpose of the decision support system
- system characteristics: mathematical model used, International Medical Device Regulators Forum (IMDRF) risk classification, type of support, , attempts to increase the interpretability of the model
- metrics of human performance: type and value of all the metrics used to assess human performance with and without the support system, including, but not limited to, sensitivity, specificity, area under the receiver operating characteristic curve (AUC), positive and negative predictive values, precision, accuracy, recall, position, time to decision, inter- and intra-operator variability, usability, clinical outcomes (mortality, morbidity, adverse events) and institutional outputs (average cost of treatment, length of stay)
- metrics of computer performance: type and value of all the metrics used to assess the performance of the decision support system alone, including, but not limited to, sensitivity, specificity, AUC, positive and negative predictive values, precision, accuracy, recall, position and time to decision.
- study funding: provenance
- existence of a published study protocol”

26.11.19: One exclusion criterion has been added to strengthen the theoretical approach.

- describing decision support systems based on natural language processing only

16.03.20: The time period considered for inclusion was reduced to 01.01.2010 – 31.05.19 This change was decided for the following reasons. I) The nomenclature used to describe the publications of interest has evolved over time and using the described search strategy over an unrestricted period of time would only yield a partial coverage. II) Several publications describe only the commercial names of the systems tested. With increasing elapsed time since publication, it becomes more and more difficult to contact the authors or the manufacturers to

obtain details critical to assess inclusion criteria. III) It is common practice in the field to limit the search to the last few years.
This change was made based on observations obtained during the full text screening phase and before any data extraction started.

Old: “Years: 1806 (PsycINFO) / 1946 (Medline) / 1974 (Embase) to 31.05.2019.”

New: “01.01.2010 to 31.05.19”

16.03.20: The assessment of bias strategy was updated to better reflect the specificity of the included publications.

Old: “The risk of bias in individual studies will be assess using the QUADAS-2 tool modified after Riches. QUADAS-2 was developed to assess the risk of bias in studies investigating diagnostic tests and is recommended by by the National Institute for Health and Care Excellence (NICE) and the Agency for Healthcare Regulation and Quality (AHRQ).
The tool assesses four different components of the study design independently (patient selection, index test, reference standard, and flow and timing) and does not allow an overall score to be calculated. Riches et al. extended the QUADAS-2 tool by including the source of funding in the bias assessment. A summary of the assessment will be included in the systematic review.”

New: “The risk of bias in individual studies will be assess using the QUADAS-2 tool modified after Riches. QUADAS-2 was developed to assess the risk of bias in studies investigating diagnostic tests and is recommended by the National Institute for Health and Care Excellence (NICE) and the Agency for Healthcare Regulation and Quality (AHRQ).
The tool assesses four different components of the study design independently (patient selection, index test, reference standard, and flow and timing) and does not allow an overall score to be calculated. Riches et al. extended the QUADAS-2 tool by including the source of funding in the bias assessment.
The subsections and signalling questions from the ROBIN-I assessment tool applicable to the included studies will also be used to complement the risk of bias assessment. This reflects the complex nature of the included studies, evaluating both the performance of a diagnostic test and of an intervention on physicians.
A summary of the assessment will be included in the systematic review.”

INTRODUCTION

Rationale

The last decade has seen an exponential growth in the number of computational tools using large sets of patient data routinely collected in healthcare settings to perform clinical decision tasks. Previously the prerogative of human physicians, these tasks range from tumour classification to outcome prediction, via radiological diagnostics and triage.

The vast majority of these computational tools are tested for efficiency on specifically designated test datasets or against humans as reference standards, but rarely for the benefit they can have when used as adjunct to clinicians’ decision-making. It is unlikely that human physicians will disappear from the medical decision-making process in the near future[3] and, as long as the responsibility and liability for patient care remains with them, the human perception of a problem and decision regarding the solution will be crucial factors influencing patient outcomes. Hence, it is important to understand the effects on human performance of this new generation of decision support systems using machine learning algorithms and based on patient data.

Computerized decision support systems are not new in medicine and have already been the subjects of numerous systematic reviews.[4–7] However, the recent advances in computer sciences have opened the door to a new class of clinical algorithms, which, unlike their predecessors, are not building their recommendations on handcrafted knowledge bases but on their own interpretation of thousands if not millions of data points derived from agnostic clinical data. While this novelty offers the opportunity of increased accuracy and relevance, it also introduces new obstacles related to the interpretability and reliability of the software’s outputs. By design these algorithms have the potential to outperform their human operators so that the human contribution can become the limiting factor. The notion of trust and the need to understand how recommendations were produced play a crucial role in bridging the software outputs to actual effects on patient outcomes.

Moreover, the usability of a system and its seamless integration into the clinical workflow are important considerations toward a broad deployment of this technology and translating its benefits into improved patient care.

Understanding the impact of specific software design components, like the mathematical approach or the degree of support provided, on the human perception of the system's abilities and access to appropriate metrics to evaluate the non-technical aspects of the human-computer interaction would be useful to orient the development and testing of future decision support systems based on machine learning.

Objectives

The primary objective of this systematic review is to evaluate the effects of clinical decision support systems based on machine learning on physicians' performance, focusing on diagnosis or diagnostic investigations planning.

Secondary objectives are:

- To compare the performance of the human-computer interactions to the performance of the computer systems alone.
- To identify the evaluation metrics commonly used to evaluate human-computer interaction and performance in the context of medical diagnostic based on machine learning.
- To identify potential gaps in the assessment methodology of human-computer interactions in the context of medical diagnostic based on machine learning.
- To assess if particular strategies for decision support systems' design (mathematical approach, degree of support, timing of support, etc) are consistently associated with better physicians' performance.

METHODS

Eligibility criteria (all should be met)

Study types:	This systematic review will focus on primary research only. This can include, but is not limited to, randomized control trials, case-control trials, cohort studies, before and after studies as well as qualitative research. Case reports and case series will be excluded.
Years:	01.01.2010 to 31.05.2019
Language:	English literature only
Population:	Human medical doctors from all specialties and all levels of seniority, in both in- and outpatient settings, facing a clinical diagnostic decision having a direct impact on patient care. Medical students are not included in the study population.
Intervention:	Interactive use of a decision support system based on clinical data and machine learning algorithms to improve diagnosis or diagnostic investigations planning. In the context of this review, machine learning algorithms are defined as algorithms that have the ability to independently learn, from clinical data, knowledge unknown to their programmers and to generate outputs that have not been explicitly programmed. Machine learning models considered as general medical statistics, such as linear regression and logistic regression are not included. Diagnosis is defined as "the identification of the nature of an illness" (Oxford Dictionary).
Control:	Human medical doctors without the aforementioned decision support system. This includes studies where the same individuals had to perform a task with and without the decision support system.
Outcomes:	Any metrics assessing performance, usability, trust or other components of human-computer interaction.

© 2021 Vasey B et al. *JAMA Network Open.*

Exclusion criteria: Will be excluded studies:

- only comparing the outputs of an automated system against human performance without decision support as gold standard
- describing monitoring or alert systems (including follow up monitoring)
- describing decision support systems based on handcrafted knowledge or rules bases only (human expert knowledge)
- describing decision support systems based on natural language processing only
- describing decision support systems based on validated clinical scores only
- describing systems uniquely designed to improve the quality of a signal
- whose target patients are not human

Information sources

The search strategy mentioned in hereafter will be run in Embase (without conference abstracts), Medline and PsycINFO.

Grey literature search will include: The World Health Organization International Clinical Trials Registry Platform, conference abstracts (from 2017 onward), the Cochrane Central Register of Controlled Trials.

Web of Science will be used for forward and backward literature search from included studies.

Search strategy

The following search strategy was developed with the support of an experienced librarian (TP). The initial search has been run on 20.05.19 in MEDLINE and EMBASE and on 12.06.19 in PsycINFO using the Ovid interface. The search will be repeated towards the end of the review process to make sure late indexation are also considered.

As several clinical algorithms are referred to under their trade names in the literature, and might therefore escape our search strategy, trade names will be used in addition to generic search terms to enhance the retrieval where appropriate. These studies will be included as “other resources” in the PRISMA diagram.

1: *Decision Making, Computer-Assisted/

2: exp Diagnosis, Computer-Assisted/

3: *Therapy, Computer-Assisted/

4: Drug Therapy, Computer-Assisted/

5: exp Decision Support Systems, Clinical/

6: *Algorithms/

7: (CDSS* or CCDSS* or "decision support" or "decision making" or "diagnos* support" or "computer aided" or CAD* or "computer assisted" or "digital assistance" or algorithm*).ab,kw,ti.

8: 1 or 2 or 3 or 4 or 5 or 6 or 7

9: exp Artificial Intelligence/

10: exp Latent Class Analysis/

11: exp Pattern Recognition, Automated/

12: ("artificial intelligence" or AI or "machine learning" or "deep learning" or "neural network" or "support vector machine" or "Bayesian network" or "nearest neighbour" or "decision tree" or "random forest" or "patient similarity" or "pattern recognition" or "natural language processing" or (supervised adj2 learning) or (unsupervised adj2 learning) or "reinforcement learning").ab,kw,ti.

13: 9 or 10 or 11 or 12

14: (doctor* or residen* or physician* or clinician* or surgeon* or registrar* or "house officer*" or fellow* or medics or consultant* or attending or practitioner* or oncologist* or pathologist* or radiologist* or ophthalmologist* or neurologist* or cardiologist* or urologist* or gynecologist* or gastroenterologist* or pneumologist* or dermatologist* or endocrinologist* or psychiatrist* or pediatrician* or internist* or anesthesiologist* or orthopedist*).ab,kw,ti.

15: (safety or trust or usability or confidence or reliability or performance or outperform* or metrics or measure* or evaluat* or assess* or effective* or precision or recall or accuracy or "patient* outcome*" or "clinical outcome*" or "surgical outcome*" or "term outcome*" or mortality or morbidity or complication*).ab,kw,ti.

16: 8 and 13 and 14 and 15

17: limit 16 to (editorial or letter or "review" or "systematic review")

18: 16 not 17

Study records

Data management: Deduplication will be carried out both automatically and manually using the EndNote software. The abstracts screening, study selection and data extraction will be performed with the Covidence systematic review online tool.[8]

Selection process: Abstracts screening will be performed by at least two independent reviewers. The first reviewer will screen through all of the abstracts. Conflicts will be resolved by a third reviewer. Abstracts meeting the inclusion criteria or possibly meeting the inclusion criteria will be selected for full text review and pdf files will be uploaded to the systematic review library.

Full text review and inclusion will be performed by at least two reviewers. The first reviewer will review all the selected publications. Conflicts will be resolved by discussion and a third reviewer will adjudicate any unresolved conflict.

Data collection process: Data extraction and collection will be performed by at least two independent reviewers. Data will be collected using a standardised extraction sheet designed by the first reviewer and containing all the items mentioned in Item 12. Reviewers will attend a practical introduction to ensure consistency of the data collection. Conflicts will be resolved by discussion and a third reviewer will adjudicate any unresolved conflict.

Given the expected high heterogeneity of measured outcomes, authors will not necessarily be contacted to obtain missing data.

Data items

The following data will be extracted if present:
- study population: number, specialty, seniority
- patient population: type of medical conditions, number of different hospital sites
- dataset: type of sample, sample size and number of events (for training and validation sets), independence of training and test sets

© 2021 Vasey B et al. *JAMA Network Open.*

- experiment: task to be performed, experimental design, number of cases per physician, timing of support, gold standard comparison, familiarity with the system.
- main purpose of the decision support system
- system characteristics: mathematical model used, International Medical Device Regulators Forum (IMDRF) risk classification, type of support, attempts to increase the interpretability of the model
- metrics of human performance: type and value of all the metrics used to assess human performance with and without the support system, including, but not limited to, sensitivity, specificity, area under the receiver operating characteristic curve (AUC), positive and negative predictive values, precision, accuracy, recall, position, time to decision, inter- and intra-operator variability, usability, clinical outcomes (mortality, morbidity, adverse events) and institutional outputs (average cost of treatment, length of stay)
- metrics of computer performance: type and value of all the metrics used to assess the performance of the decision support system alone, including, but not limited to, sensitivity, specificity, AUC, positive and negative predictive values, precision, accuracy, recall, position and time to decision.
- study funding: provenance
- existence of a published study protocol

Outcomes and prioritization

The main outcome is the physicians' performance with and without the described decision support systems. We expect the metrics used to quantify performance to vary depending on the main purpose of the decision support system described. These metrics include, but are not limited to, sensitivity, specificity, AUC, positive and negative predictive values, precision, accuracy, recall, position, time to decision, inter- and intra-operator variability, usability, clinical outcomes (mortality, morbidity, adverse events) and institutional outputs (average cost of treatment, length of stay). Qualitative performance assessment will also be considered.

The additional outcomes are:

- the performance of the computer system alone. We expect the metrics used to quantify performance to vary depending on the main purpose of the decision support system. The metrics include, but are not limited to, sensitivity, specificity, AUC, positive and negative predictive values, precision, accuracy, recall, position and time to decision.
- the qualitative and quantitative evaluation of the decision support system by the human operators. We expect that only few studies address this point and the metrics used for the evaluation to be heterogenous.

Risk of bias in individual studies

The risk of bias in individual studies will be assess using the QUADAS-2 tool[9] modified after Riches.[4] QUADAS-2 was developed to assess the risk of bias in studies investigating diagnostic tests and is recommended by the National Institute for Health and Care Excellence (NICE) and the Agency for Healthcare Regulation and Quality (AHRQ).
The tool assesses four different components of the study design independently (patient selection, index test, reference standard, and flow and timing) and does not allow an overall score to be calculated. Riches et al. extended the QUADAS-2 tool by including the source of funding in the bias assessment. The subsections and signalling questions from the ROBINS-I assessment tool[10] applicable to the included studies will also be used to complement the risk of bias assessment. This reflects the complex nature of the included studies, evaluating both the performance of a diagnostic test and of an intervention on physicians.
A summary of the assessment will be included in the systematic review.

Data synthesis

Given the expected heterogeneity of the systematic review's target studies, the authors do not plan a meta-analysis at the time of registering this protocol.
A narrative synthesis of the reported outcomes in line with the systematic review's objectives will be performed, including differences in performance between the intervention and control groups as well as between the intervention group and the computer system alone. Underlying factors possibly explaining changes in effect size or direction will be investigated. The authors expect a noticeable variability in the metrics used to assess

© 2021 Vasey B et al. *JAMA Network Open.*

performance. A summary table of these metrics will be presented. Qualitative data will be presented descriptively as recommended in the PRISMA elaboration and explanation document.
Subgroups analysis will be performed according to the mathematical model used, the degree of support and the physicians' level of seniority. Any other coherent groups emerging from the included studies could also be subject to a subgroup analysis. If a subgroup is sufficiently homogenous in term of study population and performance metrics, a quantitative synthesis of the performance metrics will be considered. The minimal number of studies required for this synthesis will depend on the number of participants in each study.

Meta-bias

The Clinical Trial Register at the International Clinical Trials Registry Platform of the World Health Organisation will be searched to look for unpublished trials (publication bias) or partial reporting of outcomes (outcome reporting bias). Due to the expected heterogeneity of the systematic review's target studies, the authors do not plan to perform funnel plots.

The overall provenance of funding will also be considered in the assessment of meta-bias.

Confidence in cumulative evidence

If quantitative summary statistics are performed, the confidence in cumulative evidence will be assess according to the Grading of Recommendations Assessment, Development and Evaluation (GRADE) methodology.[11]

REFERENCES

1. Moher D, Shamseer L, Clarke M, Ghersi D, Liberati A, Petticrew M, et al. Preferred reporting items for systematic review and meta-analysis protocols (PRISMA-P) 2015 statement. Syst Rev [Internet]. 2015;4(1):1. Available from: https://doi.org/10.1186/2046-4053-4-1
2. Shamseer L, Moher D, Clarke M, Ghersi D, Liberati A, Petticrew M, et al. Preferred reporting items for systematic review and meta-analysis protocols (PRISMA-P) 2015: elaboration and explanation. BMJ Br Med J [Internet]. 2015 Jan 2;349:g7647. Available from: http://www.bmj.com/content/349/bmj.g7647.abstract
3. Topol EJ. High-performance medicine: the convergence of human and artificial intelligence. Nat Med [Internet]. 2019;25(1):44–56. Available from: https://doi.org/10.1038/s41591-018-0300-7
4. Riches N, Panagioti M, Alam R, Cheraghi-Sohi S, Campbell S, Esmail A, et al. The Effectiveness of Electronic Differential Diagnoses (DDX) Generators: A Systematic Review and Meta-Analysis. PLoS One [Internet]. 2016;11(3):1–26. Available from: https://doi.org/10.1371/journal.pone.0148991
5. Jaspers MWM, Smeulers M, Vermeulen H, Peute LW. Effects of clinical decision-support systems on practitioner performance and patient outcomes: a synthesis of high-quality systematic review findings. J Am Med Inform Assoc. 2011 May;18(3):327–34.
6. AX G, NJ A, McDonald H, al et. Effects of computerized clinical decision support systems on practitioner performance and patient outcomes: A systematic review. JAMA [Internet]. 2005 Mar 9;293(10):1223–38. Available from: http://dx.doi.org/10.1001/jama.293.10.1223
7. Bright TJ, Wong A, Dhurjati R, Bristow E, Bastian L, Coeytaux RR, et al. Effect of clinical decision-support systems: a systematic review. Ann Intern Med. 2012 Jul;157(1):29–43.
8. Covidence systematic review software, Veritas Health Innovation, Melbourne, Australia. Available at www.covidence.org.
9. Whiting PF, Rutjes AWS, Westwood ME, Mallett S, Deeks JJ, Reitsma JB, et al. QUADAS-2: a revised tool for the quality assessment of diagnostic accuracy studies. Ann Intern Med. 2011 Oct;155(8):529–36.
10. Sterne JAC, Hernán MA, Reeves BC, Savović J, Berkman ND, Viswanathan M, et al. ROBINS-I: a tool for assessing risk of bias in non-randomised studies of interventions. BMJ [Internet]. 2016 Oct 12;355:i4919. Available from: http://www.bmj.com/content/355/bmj.i4919.abstract
11. Guyatt GH, Oxman AD, Vist GE, Kunz R, Falck-Ytter Y, Alonso-Coello P, et al. GRADE: an emerging consensus on rating quality of evidence and strength of recommendations. BMJ [Internet]. 2008 Apr 24;336(7650):924 LP – 926. Available from: http://www.bmj.com/content/336/7650/924.abstract

© 2021 Vasey B et al. *JAMA Network Open.*

eAppendix 2. Modified search strategies

CONFERENCE ABSTRACTS

1: *Decision Making, Computer-Assisted/

2: exp Diagnosis, Computer-Assisted/

3: *Therapy, Computer-Assisted/

4: Drug Therapy, Computer-Assisted/

5: exp Decision Support Systems, Clinical/

6: *Algorithms/

7: (CDSS* or CCDSS* or "decision support" or "decision making" or "diagnos* support" or "computer aided" or CAD* or "computer assisted" or "digital assistance" or algorithm*).ab,kw,ti.

8: 1 or 2 or 3 or 4 or 5 or 6 or 7

9: exp Artificial Intelligence/

10: exp Latent Class Analysis/

11: exp Pattern Recognition, Automated/

12: ("artificial intelligence" or AI or "machine learning" or "deep learning" or "neural network" or "support vector machine" or "Bayesian network" or "nearest neighbour" or "decision tree" or "random forest" or "patient similarity" or "pattern recognition" or "natural language processing" or (supervised adj2 learning) or (unsupervised adj2 learning) or "reinforcement learning").ab,kw,ti.

13: 9 or 10 or 11 or 12

14: (doctor* or residen* or physician* or clinician* or surgeon* or registrar* or "house officer*" or fellow* or medics or consultant* or attending or practitioner* or oncologist* or pathologist* or radiologist* or ophthalmologist* or neurologist* or cardiologist* or urologist* or gynecologist* or gastroenterologist* or pneumologist* or dermatologist* or endocrinologist* or psychiatrist* or pediatrician* or internist* or anesthesiologist* or orthopedist*).ab,kw,ti.

15: (safety or trust or usability or confidence or reliability or performance or outperform* or metrics or measure* or evaluat* or assess* or effective* or precision or recall or accuracy or "patient* outcome*" or "clinical outcome*" or "surgical outcome*" or "term outcome*" or mortality or morbidity or complication*).ab,kw,ti.

16: 8 and 13 and 14 and 15

17: limit 16 to (editorial or letter or "review" or "systematic review")

18: 16 not 17

19: limit 18 to (conference abstracts and yr = “2017-2019”)

COCHRANE CENTRAL REGISTER OF CONTROLLED TRIALS (CENTRAL)

#1: MeSH descriptor: [Decision Making, Computer-Assisted] this term only

#2: MeSH descriptor: [Diagnosis, Computer-Assisted] explode all trees

#3: MeSH descriptor: [Therapy, Computer-Assisted] this term only

#4: MeSH descriptor: [Drug Therapy, Computer-Assisted] explode all trees

#5: MeSH descriptor: [Decision Support Systems, Clinical] explode all trees

#6: MeSH descriptor: [Algorithms] this term only

#7: CDSS* or CCDSS* or "decision support" or "decision making" or "diagnos* support" or "computer aided" or CAD* or "computer assisted" or "digital assistance" or algorithm*

#8: #1 or #2 or #3 or #4 or #5 or #6 or #7

#9: MeSH descriptor: [Artificial Intelligence] explode all trees

#10: MeSH descriptor: [Latent Class Analysis] explode all trees

#11: MeSH descriptor: [Pattern Recognition, Automated] explode all trees

#12: "artificial intelligence" or AI or "machine learning" or "deep learning" or "neural network" or "support vector machine" or "Bayesian network" or "nearest neighbour" or "decision tree" or "random forest" or "patient similarity" or "pattern recognition" or "natural language processing" or (supervised adj2 learning) or (unsupervised adj2 learning) or "reinforcement learning"

#13: #9 or #10 or #11 or #12

#14: doctor* or residen* or physician* or clinician* or surgeon* or registrar* or "house officer*" or fellow* or medics or consultant* or attending or practitioner* or oncologist* or pathologist* or radiologist* or ophthalmologist* or neurologist* or cardiologist* or urologist* or gynecologist* or gastroenterologist* or pneumologist* or dermatologist* or endocrinologist* or psychiatrist* or pediatrician* or internist* or anesthesiologist* or orthopedist*

#15: safety or trust or usability or confidence or reliability or performance or outperform* or metrics or measure* or evaluat* or assess* or effective* or precision or recall or accuracy or "patient* outcome*" or "clinical outcome*" or "surgical outcome*" or "term outcome*" or mortality or morbidity or complication*

#16: #8 and #13 and #14 and #15

#17: limit #16 to date from Jan 2010 to May 2019

WORLD HEALTH ORGANIZATION INTERNATIONAL CLINICAL TRIALS REGISTRY PLATFORM (ICTRP)

artificial intelligence and CDSS or artificial intelligence and decision support or artificial intelligence and CAD or machine learning and CDSS or machine learning and decision support or machine learning and CAD or deep learning and CDSS or deep learning and decision support or deep learning and CAD or algorithm* and CDSS or algorithm* and decision support or algorithm* and CAD

© 2021 Vasey B et al. *JAMA Network Open.*

eTable 1. metrics used to evaluate the impact of ML-based CDSS on human performance

Metric used	Occurrence	Metric used	Occurrence
Sensitivity/detection rate or number	30	True positive fraction for a given false positive fraction's interval	1
Specificity/number of false positive	26	Positive predictive value at x % prevalence	1
Area under the curve (ROC, JAFROC)	19	Negative predictive value at x % prevalence	1
Accuracy (binary, standard deviation or percentual scoring error)	14	Subjective "obviousness score"	1
Interobserver agreement/variability (Kappa, Kendall's tau, ICC, Blant & Altman, standard deviation of estimates)	11	Accuracy (multi-reader congruent diagnosis)	1
Positive predictive value	11	Sensitivity (multi-reader congruent diagnosis)	1
Negative predictive value	11	Specificity (multi-reader congruent diagnosis)	1
Reading time/time to decision in second	8	Failure to detect at least one nodule	1
Rate/number of patients recalled for further investigations	4	Detection of at least one false positive	1
Positive predictive value of further investigations	3	Confidence	1
Correct clinical management	1	Severity stratification	1
Lesion stage/class (radiological, pathological or clinical)	2	Overestimates	1
Number of discarded computer flag	1	Underestimates	1
Diagnostic odd ratio	1	Change in recommended action	1
% of a specific subgroup amongst the diagnosed lesions	1	Complete agreement of management recommendations	1

The number of occurrences represent the number of studies using the metric for at least one analysis. ROC = receiver operating characteristic, JAFROC = jackknife free-response receiver operating characteristic.

© 2021 Vasey B et al. *JAMA Network Open.*

eTable 2. Impact of ML-based CDSS on clinician performance in patients or lesions subgroup

Metrics categories	Results reported with statistical significance			Results reported without statistical significance			Total subgroup analyses
	Increase overall or for ≥ 50% of the participants	No change or unclear change as a group	Decrease overall or for ≥ 50% of the participants	Increase overall or for ≥ 50% of the participants	No change or unclear change as a group	Decrease overall or for ≥ 50% of the participants	
Sensitivity	10	15	1	3	0	1	30
Specificity	1	3	1	2	0	0	7
Area under the curve	5	1	0	2	0	0	8
Accuracy	4	5	0	1	1	0	11
Interobserver agreement	1	1	0	4	0	0	6
Positive predictive value	0	0	0	3	0	0	3
Negative predictive value	0	0	0	2	0	0	2
Reading time	0	2	2	0	3	0	7
recall for further investigations	0	3	0	0	0	0	3
PPV of further investigations	0	2	0	0	0	0	2

Number of subgroup analyses reported for the ten most commonly used metrics groups, comparing computer-assisted clinicians to clinicians alone. PPV = positive predictive value.

© 2021 Vasey B et al. *JAMA Network Open.*

eTable 3. Complete List of the Included Studies' Results for the Primary Outcome

First author	Year	Gold standard comparison	Subgroup*	Outcome	Human alone performance	Assisted human performance	Statistical significance
Aissa	2018	1 radiologist (detection)<br>2 radiologists (follow up)<br>all also study subjects	all participants together (3)	number of solid nodules detected	326	458	yes
				number of true positive nodules detected	326	418	yes
				number of ground glass opacities detected	25	8	yes
Aslantas	2016	one experienced physician	all participants together (1)	accuracy in %	95.38	96.9	NA
				sensitivity in %	97.95	98	NA
				specificity in %	87.5	90.6	NA
Bargallo	2014	positive biopsy results for the positive cases,<br>no information for the negative cases	all participants together (9 without CDSS, 4 with CDSS)	recall rate in %	3.94	7.02	NA
				biopsy rate in %	0.9	1.02	NA
				cancer detection rate in ‰	5.25	6.1	NA
				PPV of recall in %	13.32	8.69	NA
				breast cancer stage at diagnosis	0: 25.3%,<br>I: 52.6%,<br>II: 15.8%,<br>III: 3.2%,<br>IV: 1.6%,<br>NA: 0.8%	0: 21.5%,<br>I: 55.4%,<br>II: 17.7%,<br>III: 3.1%,<br>IV: 0%,<br>NA: 2.3%	NA
Barinov	2019	pathology results after biopsy or 1 year follow-up	1 radiologist with 20+ years experience	AUROC (second reader)	0.76	0.79	no
				AUROC (first reader)	0.76	0.82	yes
				sensitivity in % (first reader, OPS)	97.5	98.2	NA
				specificity in % (first reader, OPS)	62	55	NA
			1 radiologist with 10+ years experience	AUROC (second reader)	0.75	0.77	no
				AUROC (first reader)	0.75	0.83	yes
				sensitivity in % (first reader, OPS)	95.9	98.2	NA
				specificity in % (first reader, OPS)	59	47.5	NA
			1 radiologist with 5+ years experience	AUROC (second reader)	0.73	0.79	no
				AUROC (first reader)	0.73	0.8	yes
				sensitivity in % (first reader, OPS)	92.4	97	NA
				specificity in % (first reader, OPS)	54.5	53.5	NA
			all participants together (3)	inter-reader variability, Kendall's tau b (second reader)	0.42-0.55	0.56-0.66	yes
				inter-reader variability, Kendall's tau b (first reader)	0.42-0.55	0.62-0.75	yes
Bartolotta	2018	core-biopsy or 24 months follow-up	2 radiologists with 20+ years experience	cases correctly classified	257	273	no
				sensitivity in %	91.8	97.5	NA
				specificity in %	81.5	86.5	NA
				PPV in %	77.2	83.2	NA
				NPV in %	93.6	98.1	NA
				number of lesions in each BI-RADS class	NA	NA	no
				AUROC	0.93	0.95	no

© 2021 Vasey B et al. *JAMA Network Open.*

			2 radiology residents	res 1 - AUROC	0.85	0.88	yes
				res 2 - AUROC	0.83	0.87	yes
				intra-observer agreement - res 1, kappa	0.69	0.78	NA
				intra-observer agreement - res 2, kappa	0.69	0.81	NA
				inter-observer agreement - baseline, kappa	0.67	0.7	NA
				inter-observer agreement - 3 months, kappa	0.63	0.77	NA
Bien	2018	3 board certified MSK radiologists (consensus)	7 radiologists and 2 orthopedists	sensitivity (abnormality)	0.896	0.916	no
				sensitivity (ACL)	0.914	0.91	no
				sensitivity (meniscus)	0.776	0.831	no
				specificity (abnormality)	0.825	0.851	no
				specificity (ACL)	0.917	0.996	yes
				specificity (meniscus)	0.856	0.849	no
				accuracy (abnormality)	0.883	0.905	no
				accuracy (ACL)	0.916	0.939	no
				accuracy (meniscus)	0.815	0.836	no
				inter-rate reliability (abnormality), kappa	0.571	0.64	NA
				inter-rate reliability (ACL), kappa	0.754	0.84	NA
				inter-rate reliability (meniscus), kappa	0.526	0.621	NA
			the same 7 radiologists	sensitivity (abnormality)	0.905	0.926	no
				sensitivity (ACL)	0.906	0.902	no
				sensitivity (meniscus)	0.82	0.829	no
				specificity (abnormality)	0.844	0.864	no
				specificity (ACL)	0.933	0.977	yes
				specificity (meniscus)	0.882	0.88	no
				accuracy (abnormality)	0.894	0.916	no
				accuracy (ACL)	0.92	0.94	no
				accuracy (meniscus)	0.849	0.846	no
van den Biggelaar	2010	histopathology after surgery or 1 year follow up	all participants together (2)	sensitivity in %	84	84	no
				specificity in %	95	95	no
				PPV in %	45	44	no
				NPV in %	99	99	no
				diagnostic odd ratio	96	90	no
				number of positive cases	94	96	NA
Blackmon	2011	3 experienced radiologists using the CAD output (consensus)	all participants together (2)	sensitivity in % (patient)	84.4	92.2	no
				specificity in % (patient)	92.6	88.3	NA
				sensitivity in % (PEs total)	50	70.6	yes
				PPV in % (PEs total)	80.4	80.8	NA
				PPV in % (patient)	88.6	84.3	NA
				NPV in % (patient)	89.7	94.3	NA
				false positive PEs (per patient)	0.18	0.25	no
				accuracy in % (double detection, patient)	39.2	48.1	yes
				sensitivity in % (double detection, PEs total)	32.8	61.3	yes
				sensitivity in % (double detection, central)	84.6	92.3	no
				sensitivity in % (double detection, lobar)	81.8	90.9	no
				sensitivity in % (double detection, segmental)	28.6	58.9	yes
				sensitivity in % (double detection, subsegmental)	26.3	57.9	yes

© 2021 Vasey B et al. *JAMA Network Open.*

Cha	2018	1 radiologist with 32 years experience with access to histopathology of resected bladder	all participants together (12)	AUROC (all)	0.74	0.77	yes
				standard deviation of estimates on the % scale (all)	20.4	17,9	yes
				AUROC (easy cases)	0.81	0.84	NA
				standard deviation of estimates on the % scale (easy cases)	14.7	13.4	yes
				AUROC (difficult cases)	0.59	0.62	NA
				standard deviation of estimates on the % scale (difficult cases)	29.1	24.7	yes
Chabi	2012	cytology and/or pathology for all lesions BI-RADS >2	1 radiologist with 20 years experience	sensitivity in % (benign/malignant)	99	99	no
				specificity in % (benign/malignant)	70	46	yes
				sensitivity in % (BI-RADS >=4)	100	100	no
				specificity in % (BI-RADS >=4)	48	31	yes
			1 radiologist with 5 years experience	sensitivity in % (benign/malignant)	87	96	yes
				specificity in % (benign/malignant)	80	58	yes
				sensitivity in % (BI-RADS >=4)	87	96	yes
				specificity in % (BI-RADS >=4)	80	58	yes
			1 radiologist with 1 year experience	sensitivity in % (benign/malignant)	88	95	no
				specificity in % (benign/malignant)	69	57	no
				sensitivity in % (BI-RADS >=4)	97	95	no
				specificity in % (BI-RADS >=4)	34	54	yes
			1 radiologist with 4 months experience	sensitivity in % (benign/malignant)	88	91	no
				specificity in % (benign/malignant)	71	71	no
				sensitivity in % (BI-RADS >=4)	95	95	no
				specificity in % (BI-RADS >=4)	59	53	no
Cho	2017	histopathology after needle biopsy, operative resection or 2 years follow up	1 radiologist with 7 year experience	sensitivity in %	94.4	87	no
				specificity in %	49.2	86.2	yes
				PPV in %	60.7	83.9	yes
				NPV in %	91.4	88.9	no
				accuracy in %	69.8	86.6	yes
				AUROC	0.887	0.895	no
			1 radiologist with 1 year experience	sensitivity in %	94.4	83.3	yes
				specificity in %	55.4	87.7	yes
				PPV in %	63.8	84.9	yes
				NPV in %	92.3	86.4	no
				accuracy in %	73.1	85.7	yes
				AUROC	0.901	0.901	no
Choi J.-H.	2018	histopathology or 2 years follow up	2 radiologists with 5 years experience	sensitivity in %	91.7	91.7	no
				specificity in %	76.6	80.3	no
				PPV in %	20	22	yes
				NPV in %	99.3	99.3	no
				accuracy in %	77	81	no
				AUROC	0.84	0.86	no
			2 radiologists with 1 week of training	sensitivity in %	75	83.3	no
				specificity in %	71.8	77.1	no
				PPV in %	14.5	18.9	yes
				NPV in %	97.8	98.6	no
				accuracy in %	72	77.5	no
				AUROC	0.73	0.8	no

© 2021 Vasey B et al. *JAMA Network Open.*

			all participants together (4)	inter-observer agreement, kappa	0.337	0.457	yes
Choi J. S.	2019	histopathology for all lesions BI-RADS >3, or 3 with palpable mass, or growing, or on patient request, stable imaging in follow up for the rest	2 radiologists with 11 and 3 years experience	Rad 1 - sensitivity in %	88.8	86.3	no
				Rad 1 - specificity in %	72.8	93.1	yes
				Rad 1 - accuracy in %	77.9	90.9	yes
				Rad 1 - PPV in %	60.2	85.2	yes
				Rad 1 - NPV in %	93.3	93.6	no
				Rad 1 - AUROC	0.884	0.919	yes
				Rad 2 - sensitivity in %	86.3	90	no
				Rad 2 - specificity in %	83.2	90.2	yes
				Rad 2 - accuracy in %	84.2	90.1	yes
				Rad 2 - PPV in %	70.4	80.1	yes
				Rad 2 - NPV in %	92.9	95.1	no
				Rad 2 - AUROC	0.919	0.942	yes
			2 radiologists with <1 year of training	Rad 3 - sensitivity in %	88.8	95	no
				Rad 3 - specificity in %	75.1	82.1	yes
				Rad 3 - accuracy in %	79.4	86.2	yes
				Rad 3 - PPV in %	62.3	71	yes
				Rad 3 - NPV in %	93.5	97.3	no
				Rad 3 - AUROC	0.906	0.951	yes
				Rad 4 - sensitivity in %	81.3	86.3	no
				Rad 4 - specificity in %	92.5	89	yes
				Rad 4 - accuracy in %	88.9	88.1	yes
				Rad 4 - PPV in %	83.3	78.4	yes
				Rad 4 - NPV in %	91.4	93.3	no
				Rad 4 AUROC	0.895	0.914	yes
			all participants together (4)	sensitivity in %	81.3-88.8	86.3-95.0	no
				specificity in %	72.8-92.5	82.1-93.1	yes
				accuracy in %	77.9-88.9	86.2-90.9	yes
				PPV in %	60.2-83.3	71.0-85.2	yes
				NPV in %	91.4-93.5	93.3-97.3	no
				AUROC	0.884–0.919	0.914–0.951	yes
				interobserver agreement, kappa	0.538-0.706	0.632-0.788	NA
Cole	2014	histopathology or 1 years follow up	all participants together (Image Checker[†], 15)	AUROC	0.71	0.72	no
				sensitivity in %	51	53	no
				specificity in %	87	86	no
			all participants together (SecondLook[†], 14)	AUROC	0.71	0.72	no
				sensitivity in %	49	51	no
				specificity in %	89	87	no
Endo	2012	histopathology after surgery or 2 years follow up for benign lesions	2 lung specialists	accuracy in % (average)	76.7	85	NA
			1 radiologist	accuracy in %	80	93.3	NA
			all participants together (3)	accuracy in % (average)	74.4	76.7	NA
Engelke	2010	2 experienced radiologists (consensus)	1 "experienced" radiologist	percentual pulmonary embolism severity index	26.75	27.14	yes
				percentual scoring errors	4.9	3.2	yes
				correct stratifications	55	56	NA
				overestimates	0	0	NA
				underestimates	3	2	NA

© 2021 Vasey B et al. *JAMA Network Open.*

			1 "experienced" radiologist	percentual pulmonary embolism severity index	25.85	27.04	yes
				percentual scoring errors	6	4	yes
				correct stratifications	55	56	NA
				overestimates	0	0	NA
				underestimates	3	2	NA
			1 "inexperienced" radiologist	percentual pulmonary embolism severity index	20.63	23.33	yes
				percentual scoring errors	37.9	27.2	yes
				correct stratifications	42	51	NA
				overestimates	0	0	NA
				underestimates	16	7	NA
			1 "inexperienced" radiologist	percentual pulmonary embolism severity index	21.2	23.24	yes
				percentual scoring errors	31.9	28.1	yes
				correct stratifications	44	49	NA
				overestimates	0	0	NA
				underestimates	14	9	NA
			2 "experienced" radiologists	blant & Altman interobserver limits of agreement	-5.45 to 3.03	-3.67 to 2.03	NA
			2 "inexperienced" radiologists	blant & Altman interobserver limits of agreement	-19.71 to 7.47	-9.49 to 5.35	NA
			all participants together (4)	sensitivity in %	77-93	84-95	yes (individually)
				interobserver agreement, kappa	0.74	0.83	yes
Giannini	2017	biopsy or PSA follow up	all participants together (3)	sensitivity in % (patient)	80.9	87.6	no
				sensitivity in % - GS = 6 (patient)	80.6	80.6	no
				sensitivity in % - GS > 6 (patient)	81.2	91.3	yes
				sensitivity in % - diameter 4-9 mm (patient)	77.8	82.2	no
				sensitivity in % - diameter >10 mm (patient)	80	95	yes
				specificity in % (patient)	75.3	78.4	no
				PPV in % (patient)	68	72.4	no
				NPV in % (patient)	85.9	90.7	no
				sensitivity in % (lesion)	70.9	74.4	no
				sensitivity in % - GS = 6 (lesion)	69.2	71.8	no
				sensitivity in % - GS > 6 (lesion)	71.8	75.6	no
				sensitivity in % - diameter 4-9 mm (lesion)	64.9	57.9	no
				sensitivity in % - diameter >10 mm (lesion)	76.7	90	yes
				reading time in second	220	60	yes
				inter-observer agreement, kappa (patient)	0.55	0.63	no
				inter-observer agreement, kappa (lesion)	0.46	0.57	no
				reader 1 - AUROC	0.84	0.85	no
				reader 2 - AUROC	0.82	0.91	yes
				reader 3 - AUROC	0.84	0.88	no
Hwang	2019	5 board-certified radiologists in each institution with 7-14 years experience and access to CT examinations	5 thoracic radiologists	AUROC	0.932	0.958	yes
				area under the JAFROC	0.907	0.938	yes
				sensitivity (average)	0.876	0.924	yes
				specificity (average)	0.946	0.948	no

© 2021 Vasey B et al. *JAMA Network Open.*

			5 board-certified radiologists	AUROC	0.896	0.939	yes
				area under the JAFROC	0.87	0.919	yes
				sensitivity (average)	0.812	0.893	yes
				specificity (average)	0.948	0.948	no
			5 non-radiologists physicians	AUROC	0.814	0.904	yes
				area under the JAFROC	0.781	0.873	yes
				sensitivity (average)	0.699	0.835	yes
				specificity (average)	0.901	0.924	yes
Lindsey	2018	label by subspecialized orthopedic surgeons (alone or consensus)	all participants together (24)	sensitivity in %	82.7	92.5	yes
				specificity in %	87.4	94.1	yes
				Relative reduction in misinterpretation	NA	-47%	NA
Park	2019	histopathology after needle biopsy or follow up	1 radiologist with 8-10 years experience	sensitivity in %	85.4	90.2	no
				specificity in %	52.5	66.1	yes
				PPV in %	55.6	64.9	yes
				NPV in %	83.8	90.7	no
				accuracy in %	66	74	yes
				AUROC (based on malignancy score)	0.856	0.907	yes
			1 radiologist with 8-10 years experience	sensitivity in %	92.7	90.2	no
				specificity in %	54.2	66.1	yes
				PPV in %	58.5	64.9	yes
				NPV in %	91.4	90.7	no
				accuracy in %	70	76	no
				AUROC (based on malignancy score)	0.889	0.904	no
			1 first year fellowship trainee	sensitivity in %	65.9	97.6	yes
				specificity in %	27.1	23.7	no
				PPV in %	38.6	47.1	yes
				NPV in %	53.3	93.3	yes
				accuracy in %	43	54	yes
				AUROC (based on malignancy score)	0.623	0.828	yes
			1 first year fellowship trainee	sensitivity in %	75.6	85.4	no
				specificity in %	50.8	66.1	yes
				PPV in %	51.7	63.6	yes
				NPV in %	75	86.7	yes
				accuracy in %	61	74	yes
				AUROC (based on malignancy score)	0.702	0.823	yes
			1 first year fellowship trainee	sensitivity in %	87.8	97.6	no
				specificity in %	27.1	30.5	no
				PPV in %	45.6	49.4	no
				NPV in %	76.2	94.7	yes
				accuracy in %	51	58	no
				AUROC (based on malignancy score)	0.759	0.839	yes
			2 radiologists with 8-10 years experience	interobserver variability, kappa (BI-RADS category)	0.26	0.51	yes
			3 first year fellowship trainees	interobserver variability, kappa (BI-RADS category)	0.186	0.412	yes
			all participants together (5)	interobserver variability, kappa (BI-RADS category)	0.221	0.32	yes

© 2021 Vasey B et al. *JAMA Network Open.*

Rodríguez-Ruiz	2019	1 experienced radiologist with access to histopathology or 1 year follow up	all participants together (14)	AUROC	0.87	0.89	yes
				sensitivity in %	83	86	yes
				specificity in %	77	79	no
				reading time in second	146	149	no
			50% most experienced	AUROC	0.87	0.88	no
			50% least experienced	AUROC	0.87	0.89	yes
Romero	2011	not clearly stated, probably biopsy and follow up	all participants together (2)	carcinoma detection rate in ‰ (global)	11.9	14.3	no
				carcinoma detection rate in ‰ (screening)	6.1	5.6	no
				carcinoma detection rate in ‰ (diagnostic)	28.8	31.1	no
				% of DCIS in detected cancer (screening)	21.1	36.8	no
				% of DCIS in detected cancer (diagnostic)	16.1	20	no
				detection rate of microcalcification in % (global)	26.3	68.4	yes
				% of T1 tumor (global)	88	79.8	no
				% of T1 tumor (screening)	94.7	84.2	no
				% of T1 tumor (diagnostic)	83.9	78.2	no
				biopsy rate in ‰ (global)	14.7	17.9	no
				biopsy rate in ‰ (screening)	8.3	7.6	no
				biopsy rate in ‰ (diagnostic)	33.4	37.8	no
				biopsy PPV in % (screening)	73.1	69.2	no
				biopsy PPV in % (diagnostic)	86.1	82.1	no
Samulski	2010	biopsy for malignant lesions, no information for benign lesions	4 radiologists certified in mammography	mean correct localization fraction in the false-positive fraction interval ranging from 0 to 0.1	24.9	29.3	NA
				average reading time per case in seconds	70	72.8	yes
			5 non-radiologists physicians experience in mammography	mean correct localization fraction in the false-positive fraction interval ranging from 0 to 0.1	25.2	39.2	NA
				average reading time per case in seconds	97	96.7	no
			all participants together (9)	mean correct localization fraction in the false-positive fraction interval ranging from 0 to 0.1	25.1	34.8	yes
				average reading time per case in seconds	84.7	85.9	no
Sanchez Gomez	2011	not clearly stated, probably biopsy and follow up	all participants together (6)	recall rate in %	7.2	7.6	no
				biopsy rate in %	NA	NA	no
				PPV of biopsy in %	20.23	20.23	no
				sensitivity in %	96.1	97.1	NA
				specificity in %	93.2	92.8	NA
				PPV in %	6.4	6.1	NA
				NPV in %	99.5	99.5	NA
				cancer detection rate in ‰	4.2	4.3	yes
				number of cases detected	93	94	yes
Sayres	2019	3 experienced ophthalmologists (consensus)	5 retina specialists	accuracy in % (grades)	62.3	appr. 70[¶]	NA
				accuracy in % (grades + heatmap)	62.3	appr. 66[¶]	NA
			5 general ophthalmologists	accuracy in % (grades)	46.3	appr. 58[¶]	NA
				accuracy in % (grades + heatmap)	46.3	appr. 56[¶]	NA

© 2021 Vasey B et al. *JAMA Network Open.*

			all participants together (10)	sensitivity in % (grades, average)	79.4	87.5	NA
				specificity in % (grades, average)	96.6	96.1	NA
				accuracy in % (grades)	NA	NA	yes
				sensitivity in % (grades + heatmap, average)	79.4	88.7	NA
				specificity in % (grades + heatmap, average)	96.6	95.5	NA
				accuracy in % (grades + heatmap)	NA	NA	no
				accuracy in % - algorithm correct (grades)	91.1	94.4	yes
				accuracy in % - algorithm correct (grades + heatmaps)	91.1	92.6	yes
				accuracy in % - algorithm incorrect (grades)	37	32.4	no
				accuracy in % - algorithm incorrect (grades + heatmaps)	37	33.14	no
				confidence - % cases very or extremely confident (grades)	appr. 72¶	appr. 79¶	yes
				confidence - % cases very or extremely confident (grades + heatmaps)	appr. 72¶	appr. 81¶	yes
Shimauchi	2010	histopathology	all participants together (6)	AUROC	0.8	0.84	yes
				sensitivity in %	83	88	yes
				specificity in %	50	53	no
				PPV in %	66	68	no
				NPV in %	75	83	yes
				PPV in % at 20% prevalence	39	41	NA
				PPV in % at 10% prevalence	28	29	NA
				NPV in % at 20% prevalence	92	95	NA
				NPV in % at 10% prevalence	96	98	NA
Sohns	2010	NA	1 attending	median time in s (early research)	7.47	6.8	NA
				median time in s (benign)	11.12	10.93	NA
				median time in s (malignant)	11.37	11.32	NA
			1 resident	median time in s (early research)	22.6	23.56	NA
				median time in s (benign)	26.53	28.24	NA
				median time in s (malignant)	27.54	30.43	NA
Steiner	2018	3 experienced pathologists (consensus)	all participants together (6)	sensitivity in % (micrometastasis)	83.3	91.2	yes
				sensitivity in % (macrometastasis)	appr. 96¶	appr. 95¶	no
				specificity in %	appr. 99¶	100¶	no
				time to decision in s (negative)	137	111	yes
				time to decision in s (isolated tumor cells)	145	124	no
				time to decision in s (micrometastasis)	117	61	yes
				time to decision in s (macrometastasis)	39	34	no
				subjective "obviousness score" (negative)	67.5	72	no
				subjective "obviousness score" (isolated tumor cells)	55.6	50.4	no
				subjective "obviousness score" (micrometastasis)	63.1	83.6	yes
				subjective "obviousness score" (macrometastasis)	90.1	93.1	no
Stoffel	2018	histopathology	1 radiologist (8y exp.)	AUROC	0.61	0.77	no
			1 radiologist (3y exp.)	AUROC	0.77	0.75	no
			1 radiologist (2y exp.)	AUROC	0.75	0.87	no
			1 radiology resident	AUROC	0.74	0.84	no

© 2021 Vasey B et al. *JAMA Network Open.*

Sun	2014	CT scan and successful therapy with Warfarin for 6 months<br>or thrombus found during surgery	2 radiologists described as "senior"	Rad 1 - accuracy	0.883	0.997	NA
				Rad 1 - sensitivity	0.961	0.968	NA
				Rad 1 - specificity	0.859	0.98	NA
				Rad 1 - PPV	0.68	0.938	NA
				Rad 1 - NPV	0.986	0.99	NA
				Rad 1 - AUROC	0.854	0.943	yes
				Rad 2 - accuracy	0.874	0.973	NA
				Rad 2 - sensitivity	0.955	0.984	NA
				Rad 2 - specificity	0.848	0.97	NA
				Rad 2 - PPV	0.664	0.91	NA
				Rad 2 - NPV	0.984	0.995	NA
				Rad 2 - AUROC	0.848	0.942	yes
				Rad 3 - accuracy	0.865	0.969	NA
				Rad 3 - sensitivity	0.935	0.952	NA
				Rad 3 - specificity	0.842	0.975	NA
				Rad 3 - PPV	0.65	0.922	NA
				Rad 3 - NPV	0.977	0.985	NA
				Rad 3 - AUROC	0.827	0.936	NA
			2 radiologists described as "junior"	Rad 4 - accuracy	0.803	0.962	NA
				Rad 4 - sensitivity	0.916	0.935	NA
				Rad 4 - specificity	0.768	0.97	NA
				Rad 4 - PPV	0.553	0.906	NA
				Rad 4 - NPV	0.967	0.98	NA
				Rad 4 - AUROC	0.819	0.88	NA
				Rad 5 - accuracy	0.775	0.95	NA
				Rad 5 - sensitivity	0.897	0.935	NA
				Rad 5 - specificity	0.737	0.955	NA
				Rad 5 - PPV	0.517	0.866	NA
				Rad 5 - NPV	0.958	0.979	NA
				Rad 5 - AUROC	0.821	0.86	yes
			all participants together (5)	accuracy	0.84	0.966	yes
				sensitivity	0.933	0.955	yes
				specificity	0.811	0.97	yes
				PPV	0.613	0.908	yes
				NPV	0.974	0.986	yes
				AUROC	0.834	0.932	yes
Sunwoo	2017	2 experienced neuroradiologists with access to<br>follow up studies (consensus)	2 board-certified neuroradiologists	sensitivity in % (patient)	87.3	88.7	no
				false positive per patient	0.25	0.25	NA
				reading time in s	121	57.3	NA
			2 radiology residents	sensitivity in % (patient)	67.9	76.1	yes
				false positive per patient	0.1	0.12	NA
				reading time in s	97.5	64.8	NA
			all participants together (4)	sensitivity in % (patient)	77.6	81.9	NA
				false positive per patient	0.18	0.18	NA
				reading time in s	114	72	NA
				FOM	0.87	0.9	yes

© 2021 Vasey B et al. *JAMA Network Open.*

				failure to detect at least one nodule, in % of positive cases	6.7	4.2	NA
				detection of at least one FP, in % of negative cases	5.0	4.2	NA
				accuracy in % (patient)	94.2	95.8	NA
Tang	2011	2 experienced radiologists with 10+ years experience (consensus)	2 radiologists	AUROC	0.998	0.999	NA
			2 radiology residents	AUROC	0.965	0.99	NA
			2 emergency physicians	AUROC	0.879	0.942	NA
Taylor	2018	2 experienced neurologists with access to follow-up data (local),<br>PPMI core lab team (PPMI)	1 radiologist with 5+ years experience	sensitivity (local)	appr. 0.94¶	appr. 0.94¶	NA
				specificity (local)	appr. 0.91¶	appr. 0.91¶	NA
				accuracy (local)	appr. 0.93¶	appr. 0.93¶	NA
				sensitivity (PPMI)	appr. 0.90¶	appr. 0.93¶	NA
				specificity (PPMI)	appr. 0.85¶	appr. 0.93¶	NA
				accuracy (PPMI)	appr. 0.88¶	appr. 0.93¶	NA
			1 radiologist with 5+ years experience	sensitivity (local)	appr. 0.97¶	appr. 0.94¶	NA
				specificity (local)	appr. 0.82¶	appr. 0.86¶	NA
				accuracy (local)	appr. 0.91¶	appr. 0.91¶	NA
				sensitivity (PPMI)	appr. 0.85¶	appr. 0.95¶	NA
				specificity (PPMI)	appr. 0.90¶	appr. 0.93¶	NA
				accuracy (PPMI)	appr. 0.87¶	appr. 0.94¶	NA
			all participants together (2)	inter-observer reliability (local)	appr. 0.92¶	appr. 0.96¶	NA
				inter-observer reliability (PPMI)	appr. 0.91¶	appr. 0.98¶	yes
Vassallo	2018	2 experienced radiologists with access to follow up record if needed	all participants together (3)	sensitivity in % (nodule)	65	88	yes
				sensitivity in % (patient)	75	82	yes
				specificity in % (patient)	85	82	no
				reading time in s	296	329	yes
Wanatabe	2019	2 experts mammographers with access to biopsy results (consensus)	3 mammography fellowship trained radiologists	cancer detection rate in % (average)	58.5	63.75	NA
				number of false positive recall (average)	8	7.5	NA
				number of calcification recall (average)	8.5	10.25	NA
				number of discarded computer flag (calcification)	NA	6.75	NA
				number of mass recall (average)	44	47	NA
				number of discarded computer flag (mass)	NA	10.25	NA
			4 general radiologists	cancer detection rate in % (average)	40.3	59	NA
				number of false positive recall (average)	5.7	7	NA
				number of calcification recall (average)	6	11.7	NA
				number of discarded computer flag (calcification)	NA	5.3	NA
				number of mass recall (average)	30.7	41.7	NA
				number of discarded computer flag (mass)	NA	13	NA
			all participants together (7)	cancer detection rate in % (average)	51	62	NA
				number of false positive recall (average)	7	7.3	NA
				number of calcification recall (average)	7.4	10.8	NA
				Number discarded computer flag (calcification, average)	NA	6.1	NA
				number of mass recall (average)	38	44.4	NA
				number of discarded computer flag (mass, average)	NA	11	NA
				AUROC (combined readers, case scoring)	0.76	0.81	yes

© 2021 Vasey B et al. *JAMA Network Open.*

Way	2010	biopsy, other known metastatic diseases or 2 years follow up	all participants together (6)	AUROC	0.833	0.853	yes
				AUROC with true positive fraction > 0.9	0.39	0.456	yes
				AUROC (primary cancer VS benign)	0.823	0.848	yes
				AUROC with true positive fraction > 0.9 (primary cancer VS benign)	0.338	0.415	yes
				AUROC (metastatic cancer VS benign)	0.849	0.861	no
				AUROC with true positive fraction > 0.9 (metastatic cancer VS benign subset)	0.493	0.535	yes
				change in recommended action	NA	NA	no
Zhang	2016	biopsy or 6 months follow up	5 expert radiologists	AUROC	0.843	0.896	yes
				sensitivity in %	83.5	88.8	yes
				specificity in %	75.6	76	no
				inter-observer agreement, kappa	0.36	0.457	yes
				agreement on management recommendations, cases	34	45	NA
				number of correct recommendations	27	37	NA
				mean reading time in s	16.8	21.2	yes
			5 radiology residents	AUROC	0.705	0.822	yes
				sensitivity in %	63.2	80.7	yes
				specificity in %	62.6	72.9	yes
				inter-observer agreement, kappa	0.151	0.413	yes
				agreement on management recommendations, cases	20	41	NA
				number of correct recommendations	12	30	NA
				mean reading time in s	24.5	30.6	yes
			all participants together (10)	AUROC	0.774	0.859	yes
				sensitivity in %	73.3	84.7	yes
				specificity in %	69.1	74.5	yes
				agreement on management recommendations, cases	15	31	NA
				number of correct recommendations	10	28	NA
				inter-observer agreement, kappa	0.195	0.421	yes

[*]see eTable 8 for complete description of the study participants' level of experience, [¶]estimated from graphics, [†]commercial name, NA = not available, AUROC = area under the receiver operating characteristic curve, OPS = operating point shift, PPV = positive predictive value, NPV = negative predictive value, ACL = anterior cruciate ligament, PE = pulmonary embolism, BI-RADS = breast imaging-reporting and data system, GS = Gleason score, JAFROC = jackknife free-response receiver operating characteristic, DCIS = ductal carcinoma in situ, FP = false positive, FOM = figure of merit, PPMI = Parkinson's Progression Markers Initiative

© 2021 Vasey B et al. *JAMA Network Open.*

eTable 4. Impact on clinician performance of the six ML-based CDSS evaluated in representative clinical environment

Metrics categories	Results reported with statistical significance			Results reported without statistical significance			Total CDSS evaluated
	Increase overall or for ≧ 50% of the participants	No change or unclear change as a group	Decrease overall or for ≧ 50% of the participants	Increase overall or for ≧ 50% of the participants	No change or unclear change as a group	Decrease overall or for ≧ 50% of the participants	
Sensitivity	1	3	0	2	0	0	6
Specificity	0	2	0	1	0	1	4
Area under the curve	1	1	0	0	0	0	2
Accuracy	0	2	0	0	0	0	2
Interobserver agreement	1	1	0	0	0	0	2
Positive predictive value	1	1	0	1	0	1	4
Negative predictive value	0	2	0	1	1	0	4
Reading time	0	0	0	0	0	0	0
recall for further investigations	0	2	0	1	0	0	3
PPV of further investigations	0	2	0	0	0	1	3

Number of main results reported for the ten most commonly used metrics groups, comparing computer-assisted clinicians to clinicians alone. PPV = positive predictive value.

© 2021 Vasey B et al. *JAMA Network Open.*

eTable 5. Association Between Clinicians' Level of Experience and Performance Changes When Using ML-Based CDSS.

Metrics categories	increase more for juniors	increase more for experts	no difference	decrease more for experts	decrease more for juniors	Total CDSS evaluated
Sensitivity	8	1	1	0	0	10
Specificity	4	2	0	2	2	10
Area under the curve	9	1	2	0	0	12
Accuracy	7	1	0	0	0	8
Interobserver agreement	2	0	1	0	0	3
Positive predictive value	2	1	1	0	0	4
Negative predictive value	3	0	1	0	0	4
Reading time	1	1	1	1	0	4
Recall for further investigations	0	0	0	0	0	0
PPV of further investigations	0	0	0	0	0	0

Number of main results reported for the ten most commonly used metrics groups, comparing computer-assisted clinicians to clinicians alone. PPV = positive predictive value.

© 2021 Vasey B et al. *JAMA Network Open.*

eTable 6. Impact on clinician performance of ML-based CDSS according to the reader paradigm (first reader/second reader)

Metric used	Results reported with statistical significance			Results reported without statistical significance			Total
	Increase overall or for ≧ 50% of the participants	No change or unclear change as a group	Decrease overall or for ≧ 50% of the participants	Increase overall or for ≧ 50% of the participants	No change or unclear change as a group	Decrease overall or for ≧ 50% of the participants	
MAIN RESULTS							
Sensitivity	1 / 9	3 / 8	0 / 1	4 / 5	0 / 1	0 / 0	8 / 24
Specificity	0 / 6	4 / 7	0 / 1	0 / 3	0 / 2	3 / 2	7 / 21
Area under the curve	1 / 12	3 / 4	0 / 0	0 / 1	0 / 0	0 / 0	4 / 17
Accuracy	1 / 7	2 / 2	0 / 0	0 / 6	0 / 0	0 / 0	3 / 15
Interobserver agreement	2 / 5	1 / 1	0 / 0	1 / 1	0 / 0	0 / 0	4 / 7
Positive predictive value	0 / 5	2 / 1	0 / 0	0 / 2	0 / 0	0 / 2	2 / 10
Negative predictive value	0 / 3	2 / 3	0 / 0	0 / 3	0 / 1	0 / 0	2 / 10
Reading time	0 / 2	0 / 2	2 / 0	0 / 0	1 / 0	0 / 1	3 / 5
Recall for further investigations	0 / 0	0 / 2	0 / 0	0 / 1	0 / 0	0 / 0	0 / 3
PPV of further investigations	0 / 0	0 / 2	0 / 0	0 / 0	0 / 0	0 / 1	0 / 3
SUBGROUP ANALYSES							
Sensitivity	4 / 6	10 / 5	1 / 0	0 / 3	0 / 0	0 / 1	15 / 15
Specificity	1 / 0	2 / 1	0 / 1	0 / 2	0 / 0	0 / 0	3 / 4
Area under the curve	0 / 5	0 / 1	0 / 0	0 / 2	0 / 0	0 / 0	0 / 8
Accuracy	2 / 2	5 / 0	0 / 0	0 / 1	0 / 1	0 / 0	7 / 4
Interobserver agreement	0 / 1	1 / 0	0 / 0	3 / 1	0 / 0	0 / 0	4 / 2
Positive predictive value	0 / 0	0 / 0	0 / 0	0 / 3	0 / 0	0 / 0	0 / 3
Negative predictive value	0 / 0	0 / 0	0 / 0	0 / 2	0 / 0	0 / 0	0 / 2
Reading time	0 / 0	2 / 0	2 / 0	0 / 0	3 / 0	0 / 0	7 / 0
Recall for further investigations	0 / 0	0 / 3	0 / 0	0 / 0	0 / 0	0 / 0	0 / 3
PPV of further investigations	0 / 0	0 / 2	0 / 0	0 / 0	0 / 0	0 / 0	0 / 2

. Number of main results and subgroup analyses reported for the ten most commonly used metrics groups, comparing computer-assisted clinicians to clinicians alone. PPV = positive predictive value.

© 2021 Vasey B et al. *JAMA Network Open.*

eTable 7. Impact on clinician performance of ML-based CDSS according to the mathematical model used (neural networks/other models)

Metric used	Results reported with statistical significance			Results reported without statistical significance			Total
	Increase overall or for ≧ 50% of the participants	No change or unclear change as a group	Decrease overall or for ≧ 50% of the participants	Increase overall or for ≧ 50% of the participants	No change or unclear change as a group	Decrease overall or for ≧ 50% of the participants	
MAIN RESULTS							
Sensitivity	7 / 3	9 / 2	1 / 0	9 / 0	0 / 1	0 / 0	26 / 6
Specificity	5 / 1	9 / 2	1/ 0	2 / 1	2 / 0	4 / 1	23 / 5
Area under the curve	11 / 2	6 / 1	0 / 0	1 / 0	0 / 0	0 / 0	18 / 3
Accuracy	6 / 2	4 / 0	0 / 0	3 / 3	0 / 0	0 / 0	13 / 5
Interobserver agreement	4 / 3	1 / 1	0 / 0	2 / 0	0 / 0	0 / 0	7 / 4
Positive predictive value	5 / 0	2 / 1	0 / 0	2 / 0	0 / 0	1 / 1	10 / 2
Negative predictive value	3 / 0	4 / 1	0 / 0	2 / 1	1 / 0	0 / 0	10 / 12
Reading time	0 / 2	2 / 0	1 / 1	0 / 0	1 / 0	1 / 0	5 / 3
Recall for further investigations	0 / 0	2 / 0	0 / 0	1 / 0	0 / 0	0 / 0	3 / 0
PPV of further investigations	0 / 0	2 / 0	0 / 0	0 / 0	0 / 0	1 / 0	3 / 0
SUBGROUP ANALYSES							
Sensitivity	2 / 8	7 / 8	1 / 0	2 / 1	0 / 0	0 / 1	12 / 18
Specificity	1 / 0	2 / 1	1 / 0	0 / 2	0 / 0	0 / 0	4 / 3
Area under the curve	1 / 4	0 / 1	0 / 0	2 / 0	0 / 0	0 / 0	3 / 5
Accuracy	4 / 0	5 / 0	0 / 0	0 / 1	0 / 1	0 / 0	9 / 2
Interobserver agreement	0 / 1	0 / 1	0 / 0	3 / 1	0 / 0	0 / 0	3 / 3
Positive predictive value	0 / 0	0 / 0	0 / 0	2 / 1	0 / 0	0 / 0	2 / 1
Negative predictive value	0 / 0	0 / 0	0 / 0	2 / 0	0 / 0	0 / 0	2 / 0
Reading time	0 / 0	2 / 0	2 / 0	0 / 0	3 / 0	0 / 0	7 / 0
Recall for further investigations	0 / 0	3 / 0	0 / 0	0 / 0	0 / 0	0 / 0	3 / 0
PPV of further investigations	0 / 0	2 / 0	0 / 0	0 / 0	0 / 0	0 / 0	2 / 0

Number of main results and subgroup analyses reported for the ten most commonly used metrics groups, comparing computer-assisted clinicians to clinicians alone. PPV = positive predictive value.

© 2021 Vasey B et al. *JAMA Network Open.*

eTable 8. Impact on Clinician Performance of ML-Based CDSS According to the Outputs' Level of Support (Single Output/Explanatory Output).

Metric used	Results reported with statistical significance			Results reported without statistical significance			Total
	Increase overall or for ≧ 50% of the participants	No change or unclear change as a group	Decrease overall or for ≧ 50% of the participants	Increase overall or for ≧ 50% of the participants	No change or unclear change as a group	Decrease overall or for ≧ 50% of the participants	
MAIN RESULTS							
Sensitivity	6 / 4	7/ 4	0 / 1	4 / 5	1 / 0	0 / 0	18 / 14
Specificity	1 / 5	6 / 5	1 / 0	1 / 2	2 / 0	3 / 2	14 / 14
Area under the curve	5 / 8	4 / 3	0 / 0	1/ 0	0 / 0	0 / 0	10 / 11
Accuracy	3 / 5	0 / 4	0 / 0	3 / 3	0 / 0	0 / 0	6 / 12
Interobserver agreement	4 / 3	0 / 2	0 / 0	0 / 2	0 / 0	0 / 0	4 / 7
Positive predictive value	1 / 4	1 / 2	0 / 0	1 / 1	0 / 0	2 / 0	5 / 7
Negative predictive value	1 / 2	1 / 4	0 / 0	2/ 1	1 / 0	0 / 0	5 / 7
Reading time	1 / 1	2 / 0	1 / 1	0 / 0	1 / 0	1 / 0	6 / 2
Recall for further investigations	0 / 0	2 / 0	0 / 0	1 / 0	0 / 0	0 / 0	3 / 0
PPV of further investigations	0 / 0	2 / 0	0 / 0	0 / 0	0 / 0	1 / 0	3 / 0
SUBGROUP ANALYSES							
Sensitivity	7 / 3	6 / 9	1 / 0	3 / 0	0 / 0	1 / 0	18 / 12
Specificity	0 / 1	1 / 2	1 / 0	2 / 0	0 / 0	0 / 0	4 / 3
Area under the curve	0 / 5	0 / 1	0 / 0	0 / 2	0 / 0	0 / 0	0 / 8
Accuracy	0 / 4	0 / 5	0 / 0	1 / 0	1 / 0	0 / 0	2 / 9
Interobserver agreement	1 / 0	0 / 1	0 / 0	1 / 3	0 / 0	0 / 0	2 / 4
Positive predictive value	0 / 0	0 / 0	0 / 0	1 / 2	0 / 0	0 / 0	1 / 2
Negative predictive value	0 / 0	0 / 0	0 / 0	0 / 2	0 / 0	0 / 0	0 / 2
Reading time	0 / 0	2 / 0	2 / 0	0 / 0	3 / 0	0 / 0	7 / 0
Recall for further investigations	0 / 0	3 / 0	0 / 0	0 / 0	0 / 0	0 / 0	3 / 0
PPV of further investigations	0 / 0	2 / 0	0 / 0	0 / 0	0 / 0	0 / 0	2 / 0

Number of main results and subgroup analyses reported for the ten most commonly used metrics groups, comparing computer-assisted clinicians to clinicians alone. PPV = positive predictive value.

© 2021 Vasey B et al. *JAMA Network Open.*

eTable 9. Impact of the Human Contribution on the System Performance in Patients or Lesions Subgroups

Metrics categories	Results reported with statistical significance			Results reported without statistical significance			Total subgroup analyses
	Increase overall or for ≧ 50% of the participants	No change or unclear change as a group	Decrease overall or for ≧ 50% of the participants	Increase overall or for ≧ 50% of the participants	No change or unclear change as a group	Decrease overall or for ≧ 50% of the participants	
Sensitivity	0	0	0	4	3	3	10
Specificity	0	0	0	5	1	3	9
Area under the curve	0	0	0	0	0	4	4
Accuracy	0	0	0	4	1	0	5
Interobserver agreement	0	0	0	0	0	0	0
Positive predictive value	0	0	0	1	0	0	1
Negative predictive value	0	0	0	0	0	0	0
Reading time	0	0	0	0	0	0	0
recall for further investigations	0	0	0	0	0	0	0
PPV of further investigations	0	0	0	0	0	0	0

Number of subgroup analyses reported for the ten most commonly used metrics groups, comparing computer-assisted clinicians to stand-alone computer. PPV = positive predictive value.

© 2021 Vasey B et al. *JAMA Network Open.*

eTable 10. Complete List of the Included Studies' Results for the Secondary Outcome (Assisted Human Performance vs Stand-Alone Computer Performance)

First author	Year	Same test set used to test computer performance	Study participants*	Outcome	Stand-alone computer performance	Assisted human performance	Statistical significance
Aslantas	2016	yes	1 physician	accuracy in %	92.3	96.9	NA
				sensitivity in %	94	98	NA
				specificity in %	86.67	90.6	NA
Barinov	2019	yes	3 radiologists (compared individually)	sensitivity in %	B: 100 ;<br>PB: 98;<br>S: 95;<br>M: 50	BR-2: 100;<br>BR-3: 96-100;<br>BR-4: 92-97;<br>BR-5: 19-29	NA
				specificity in %	B: 43;<br>PB: 62;<br>S: 96;<br>M: 100	BR-2: 13-20;<br>BR-3: 39-46;<br>BR-4: 98-99;<br>BR-5: 100	NA
				AUROC (second reader)	0.86	0.77-0.79	NA
				AUROC (first reader)	0.86	0.80-0.83	NA
Bien	2018	yes	7 radiologists +<br>2 orthopedists	sensitivity (abnormality)	0.879	0.916	NA
				sensitivity (ACL)	0.759	0.91	NA
				sensitivity (meniscus)	0.71	0.831	NA
				specificity (abnormality)	0.714	0.851	NA
				specificity (ACL)	0.968	0.996	NA
				specificity (meniscus)	0.741	0.849	NA
				accuracy (abnormality)	0.85	0.905	NA
				accuracy (ACL)	0.867	0.939	NA
				accuracy (meniscus)	0.725	0.836	NA
v. d. Biggelaar	2010	subset	2 radiologists	sensitivity in %	78	84	NA
Blackmon	2011	yes	2 radiologists	sensitivity (patient)	93.8	92.2	NA
				specificity (patient)	14.9	88.3	NA
				sensitivity (PEs total)	78.2	70.6	NA
				PPV (PEs total)	26.5	80.8	NA
				PPV (patient)	42.9	84.3	NA
				NPV (patient)	77.8	94.3	NA
				false positive rate PEs (per patient)	3.27	0.25	NA
Cha	2018	yes	12 physicians	AUROC	0.8	0.77	NA
				AUROC (easy cases)	0.88	0.84	NA
				AUROC (difficult cases)	0.65	0.62	NA
Chabi	2012	yes	1 radiologist with 20 years experience	sensitivity in % (benign/malignant)	100	99	NA
				specificity in % (benign/malignant)	48	46	NA
			1 radiologist with 5 years experience	sensitivity in % (benign/malignant)	100	96	NA
				specificity in % (benign/malignant)	48	58	NA
				sensitivity in % (benign/malignant)	100	95	NA

© 2021 Vasey B et al. *JAMA Network Open.*

			1 radiologist with 1 years experience	specificity in % (benign/malignant)	48	57	NA
			1 radiologist with 4 months experience	sensitivity in % (benign/malignant)	100	91	NA
				specificity in % (benign/malignant)	48	71	NA
Cho	2017	yes	1 radiologist with 7 year experience	sensitivity in %	72.2	87	NA
				specificity in %	90.8	86.2	NA
				PPV in %	86.7	83.9	NA
				NPV in %	79.7	88.9	NA
				accuracy in %	82.4	86.6	NA
				AUROC	0.815	0.895	NA
			1 radiologist with 1 year experience	sensitivity in %	72.2	83.3	NA
				specificity in %	90.8	87.7	NA
				PPV in %	86.7	84.9	NA
				NPV in %	79.7	86.4	NA
				accuracy in %	82.4	85.7	NA
				AUROC	0.815	0.901	NA
Choi J.-H.	2018	yes	2 radiologists with 5 years experience	sensitivity in %	75	91.7	NA
				specificity in %	78.2	80.3	NA
				PPV in %	18	22	NA
				NPV in %	98	99.3	NA
				accuracy in %	78	81	NA
				AUROC	0.77	0.86	NA
			2 radiologists with 1 week of training in breast imaging	sensitivity in %	66.7	83.3	NA
				specificity in %	76.1	77.1	NA
				PPV in %	15.1	18.9	NA
				NPV in %	97.3	98.6	NA
				accuracy in %	75.5	77.5	NA
				AUROC	0.71	0.8	NA
Choi J.-S	2019	yes	2 radiologists with 11 and 3 years experience	Rad 1 - sensitivity in %	85	86.3	NA
				Rad 1 - specificity in %	95.4	93.1	NA
				Rad 1 - accuracy in %	92.1	90.9	NA
				Rad 1 - PPV in %	93.2	85.2	NA
				Rad 1 - NPV in %	89.5	93.6	NA
				Rad 2 - sensitivity in %	85	90	NA
				Rad 2 - specificity in %	95.4	90.2	NA
				Rad 2 - accuracy in %	92.1	90.1	NA
				Rad 2 - PPV in %	93.2	80.1	NA
				Rad 2 - NPV in %	89.5	95.1	NA
			2 radiologists with <1 year of training in breast imaging	Rad 3 - sensitivity in %	85	95	NA
				Rad 3 - specificity in %	95.4	82.1	NA
				Rad 3 - accuracy in %	92.1	86.2	NA
				Rad 3 - PPV in %	93.2	71	NA
				Rad 3 - NPV in %	89.5	97.3	NA
				Rad 4 - sensitivity in %	85	86.3	NA
				Rad 4 - specificity in %	95.4	89	NA
				Rad 4 - accuracy in %	92.1	88.1	NA

© 2021 Vasey B et al. *JAMA Network Open.*

				Rad 4 - PPV in %	93.2	78.4	NA
				Rad 4 - NPV in %	89.5	93.3	NA
Cole	2014	yes	15 radiologists (Image Checker†)	sensitivity in %	73	53	NA
			14 radiologists (SecondLook†)	sensitivity in %	75	51	NA
Engelke	2010	yes	1 "experienced" radiologist	percentual pulmonary embolism severity index	9.85	27.14	NA
			1 "experienced" radiologist	percentual pulmonary embolism severity index	9.85	27.04	NA
			1 "inexperienced" radiologist	percentual pulmonary embolism severity index	9.85	23.33	NA
			1 "inexperienced" radiologist	percentual pulmonary embolism severity index	9.85	23.24	NA
Giannini	2017	no	3 radiologists	sensitivity in % (patient)	97	87.6	NA
Hwang	2019	yes	5 thoracic radiologists	AUROC	0.983	0.958	NA
				area under the JAFROC curve	0.985	0.938	NA
				sensitivity (high sensitivity threshold)	0.913	0.924	NA
				specificity (high sensitivity threshold)	1	0.948	NA
			5 board-certified radiologists	AUROC	0.983	0.939	NA
				area under the JAFROC curve	0.985	0.919	NA
				sensitivity (high sensitivity threshold)	0.913	0.893	NA
				specificity (high sensitivity threshold)	1	0.948	NA
			5 non-radiologists physicians	AUROC	0.983	0.904	NA
				area under the JAFROC curve	0.985	0.873	NA
				sensitivity (high sensitivity threshold)	0.913	0.835	NA
				specificity (high sensitivity threshold)	1	0.924	NA
Lindsey	2018	yes	24 emergency physicians	sensitivity in %	93.9	92.5	NA
				specificity in %	94.5	94.1	NA
Rodríguez-Ruiz	2019	yes	14 radiologists	AUROC	0.87	0.89	no
Sanchez Gomez	2011	yes	6 radiologists	sensitivity in %	84	97.1	NA
				specificity in %	13.2	92.8	NA
				PPV in %	0.46	6.1	NA
				NPV in %	99.4	99.5	NA
Sayres	2019	yes	5 retina specialists	accuracy in % (grades)	NA	NA	yes
				accuracy in % (grades + heatmaps)	NA	NA	yes
			5 general ophthalmologists	accuracy in % (grades)	NA	NA	no
				accuracy in % (grades + heatmaps)	NA	NA	no
			10 ophthalmologists	sensitivity in % (grades, average)	91.5	87.5	NA
				specificity in % (grades, average)	94.7	96.1	NA
				sensitivity in % (grades + heatmap, average)	91.5	88.7	NA
				specificity in % (grades + heatmap, average)	94.7	95.5	NA
Shimauchi	2010	yes	6 radiologists	AUROC	0.86	0.84	NA
Steiner	2018	yes	6 pathologists	sensitivity in % at 100% specificity (micrometastasis)	85	all individual pathologist performed equally or better	NA

© 2021 Vasey B et al. *JAMA Network Open.*

Stoffel	2018	yes	1 radiologist with 8 years of experience	AUROC	0.73	0.77	NA
			1 radiologist with 3 years of experience	AUROC	0.73	0.75	NA
			1 radiologist with 2 years of experience	AUROC	0.73	0.87	NA
			1 3rd year radiology resident	AUROC	0.73	0.84	NA
Sun	2014	yes	5 radiologists	accuracy	0.909	0.966	NA
				sensitivity	0.863	0.955	NA
				specificity	0.923	0.97	NA
				PPV	0.779	0.908	NA
				NPV	0.956	0.986	NA
				AUROC	0.909	0.932	NA
Sunwoo	2017	yes	4 radiologists	sensitivity in % (algorithm A)	87.3	81.9	NA
				false positive per patient (algorithm A)	302.4	0.18	NA
Taylor	2018	yes	1 radiologist with 5+ year experience	Rad 1 - sensitivity - local	appr. 0.94¶	appr. 0.94¶	NA
				Rad 1 - specificity - local	appr. 0.96¶	appr. 0.91¶	NA
				Rad 1 - accuracy - local	appr. 0.92¶	appr. 0.93¶	NA
				Rad 1 - sensitivity - PPMI	appr. 0.95¶	appr. 0.93¶	NA
				Rad 1 - specificity - PPMI	appr. 0.88¶	appr. 0.93¶	NA
				Rad 1 - accuracy - PPMI	appr. 0.92¶	appr. 0.93¶	NA
			1 radiologist with 5+ year experience	Rad 2 - sensitivity - local	appr. 0.94¶	appr. 0.94¶	NA
				Rad 2 - specificity - local	appr. 0.96¶	appr. 0.86¶	NA
				Rad 2 - accuracy - local	appr. 0.92¶	appr. 0.91¶	NA
				Rad 2 - sensitivity - PPMI	appr. 0.95¶	appr. 0.95¶	NA
				Rad 2 - specificity - PPMI	appr. 0.88¶	appr. 0.93¶	NA
				Rad 2 - accuracy - PPMI	appr. 0.92¶	appr. 0.94¶	NA
Vassallo	2018	yes	3 radiologists	sensitivity in % (nodule)	85	88	NA
Wanatabe	2019	no	7 radiologists	AUROC (individual readers)	0.66	(5/7 radiologists performed better)	NA
Way	2010	yes	6 radiologists	AUROC	0.857	0.853	NA
				AUROC with true positive fraction > 0.9	0.476	0.456	NA
Zhang	2016	yes	10 radiologists	AUROC	0.892	0.859	NA
				sensitivity in %	84.2	84.7	NA
				specificity in %	84.4	74.5	NA

*see eTable 8 for complete description of the study participants' level of experience, ¶estimated from graphics, †commercial name, NA = not available, B = benign, PB = probably benign, S = suspicious, M = malignant, BR-X= breast imaging-reporting and data system score of X, AUROC = area under the receiver operating characteristic curve, ACL = anterior cruciate ligament, PE = pulmonary embolism, PPV = positive predictive value, NPV = negative predictive value, JAFROC = jackknife free-response receiver operating characteristic, PPMI = Parkinson's Progression Markers Initiative

© 2021 Vasey B et al. *JAMA Network Open.*

eTable 11. Characteristics Relevant to the Human Factors Evaluation of the Included Studies

First author	Year	Task to be performed	Description of the CDSS' support	Attempt to increase the interpretability of the CDSS' outputs	Level of experience of the study participants	Clinicians' familiarity with the system	Attempt to gather user feedback on the system
Aissa	2018	identification (lung nodules, ground glass opacities)	marking of suspicious lesions	NA	3 resident/board-certified radiologists with 5-6 years experience	NA	NA
Aslantas	2016	classification (metastasis, no metastasis)	hotspots marking with multiple colours scale 0 (no metastasis) / 1 (metastasis) classification	heatmap	1 non-specified doctor	NA	NA
Bargallo	2014	classification (normal, recallable)	marking of suspicious lesions, shape depending on lesion characteristics	NA	4 radiologists with and without breast unit experience	one-month familiarisation period	NA
Barinov	2019	classification (BI-RADS) and score assignment (likelihood of malignancy)	4 groups classification (benign, probably benign, suspicious, malign)	NA	1 ABR certified and breast fellowship trained radiologist with 20+ years experience and 1 ABR certified and breast fellowship trained radiologist with 10 years experience and 1 ABR certified radiologist with 5 years experience	30 min training + 10 practice cases with supervision	NA
Bartolotta	2018	classification (BI-RADS)	2 groups classification (possibly benign, possibly malign)	displaying of the input features extracted/used to generate the recommendation	2 radiologists with 20+ years experience in breast US and 2 4th/5th year resident radiologists	5h training session with 20 practice cases	NA
Bien	2018	classification (normal, abnormal; ACL intact, tear; meniscus intact, tear)	4 groups classification (normal, abnormal, ACL tear, meniscal tear) and probability score	heatmap of the important features	7 board-certified general radiologists and 2 orthopaedic surgeons with together 3-29 years experience	NA	NA
van den Biggelaar	2010	sketch of the lesion and classification (BI-RADS) and prescription (additional diagnostic test)	marking of suspicious lesions, shape depending on lesion characteristics	NA	2 radiologists with 5 and 20 years experience in mammography	instruction by manufacturer and 9 months optional use	Questionnaires about the added value of the CDSS diagnostic information
Blackmon	2011	identification (suspected PE)	marking of suspicious vessels	NA	2 first year resident radiologists with 9 months experience	NA	NA
Cha	2018	score assignment (likelihood of T0 disease, % response to treatment, grade of lesion conspicuity)	complete response likelihood score	display of the CDSS-T score distribution in a graphic	9 radiologists and 2 oncologists and 1 urologist with together 2-36 years experience	NA	NA

© 2021 Vasey B et al. *JAMA Network Open.*

Chabi	2012	classification (benign, malign) and classification (BI-RADS) and score assignment (malignancy score) and characterisation (lesion type)	5 groups classification (BI-RADS 1-5)	NA	1 radiologist with 20 years experience and 1 radiologist with 5 years experience and 1 radiologist with 1 years experience and 1 radiologist with 4 months experience	NA	NA
Cho	2017	classification (BI-RADS)	2 groups classification (possibly benign, possibly malign)	displaying of the input features extracted/used to generate the recommendation	2 radiologists with 7 and 1 years experience in breast imaging	NA	NA
Choi J.-H.	2018	classification (BI-RADS)	2 groups classification (possibly benign, possibly malign)	displaying of the input features extracted/used to generate the recommendation	2 radiologists with 5 years experience in breast imaging and 2 radiologists with 1 week of training in breast imaging	NA	NA
Choi J. S.	2019	classification (BI-RADS)	2 groups classification (possibly benign, possibly malign)	displaying of the input features extracted/used to generate the recommendation	2 radiologists with 11 and 3 years experience in breast imaging and 2 radiologists with <1 year experience in breast imaging	NA	NA
Cole	2014	classification (BI-RADS) and score assignment (DMIST probability of malignancy)	Image Checker: marking of suspicious lesions SecondLook: marking of suspicious lesions, shape depending on lesion characteristics	NA	Image Checker*: 15 radiologists with 6-40 years experience in mammography Secondlook*: 14 radiologists with 3.5-32 years experience in mammography	all selected participants had clinical experience using CAD	NA
Endo	2012	classification (benign, malign)	list of 4 similar cases with diagnosis	NA	1 radiologist with unknown experience and 2 lung specialists radiologists with 7 and 10 years experience	NA	The users were invited to rate the level of similarity of the most similar case on a 1-5 scale
Engelke	2010	score assignment (Mastora risk stratification) and identification (PE)	marking of suspicious vessels	NA	2 "inexperienced" radiologists and 2 "experienced" radiologists	NA	NA
Giannini	2017	characterisation (lesion) and localisation (lesion) and classification (PI-RADS) and classification (prostate carcinoma yes, no) and score assignment (self-confidence for malignancy)	coloured malignancy likelihood map (heatmap)	per voxel malignancy likelihood map	3 radiologists with 2-4 years experience in prostate MRI	NA	NA

Hwang	2019	classification (significant findings requiring treatment, no significant findings)	localisation probability for each disease (heatmap) and overall probability of abnormal findings	per-pixel disease probability per disease visualisation	5 specialised thoracic radiologists with 9-14 years experience and 5 board certified radiologists with 5-7 years experience and 5 non-radiologist physicians of unknown experience	NA	NA
Lindsey	2018	classification (fracture present, not present)	Fracture probability value and dense conditional probability map (heatmap)	per pixel confidence in fracture probability value	24 emergency physicians of unknown experience	NA	NA
Park	2019	classification (BI-RADS) and score assignment (malignancy)	2 groups classification (possibly benign, possibly malign)	displaying of the input features extracted/used to generate the recommendation	2 radiologists with 10 and 8 years experience 3 first year fellowship trainee radiologists	NA	NA
Rodríguez-Ruiz	2019	classification (BI-RADS) and score assignment (malignancy)	marking of suspicious lesions and level of suspicion score (0-100) and Transpara score (0-10)	NA	11 specialised breast radiologists and 3 general radiologists with together 3-25 years experience	45 practice cases	NA
Romero	2011	classification (normal, recallable)	marking of suspicious lesions	NA	2 specialised breast radiologists with 9 and 5 years experience	6 months familiarisation period and 3225 practice cases between the two participants	NA
Samulski	2010	score assignment (malignancy)	coloured-coded circle around the suspicious lesion if ROI is queried and malignancy score	NA	4 radiologists "certified in mammography" and 5 non-radiologists physicians "experienced in mammography"	10 to 60 practice cases per participant	informal feedback after the test
Sanchez Gomez	2011	classification (normal, recallable)	marking of suspicious lesions, shape depending on lesion characteristics	NA	2 general radiologists with 3-9 months experience in mammography and 4 specialised breast radiologists with 2-10 years experience in mammography	NA	NA
Sayres	2019	classification (DR grade) and score assignment (confidence in diagnosis)	Grades of evidence for each diabetic retinopathy category + heatmap in explanatory mode	heatmap highlighting image regions most contributing to the prediction	4 fellowship trained retina specialists and 1 retina fellow and 5 board certified ophthalmologists	briefing about the CDSS	NA

© 2021 Vasey B et al. *JAMA Network Open.*

Shimauchi	2010	score assignment (probability of malignancy) and prescription (recommended management)	contours of segmented lesion and graphical representation of estimated probability of malignancy and kinetic curves informing about signal intensity over time and display of most-enhancing regions within given lesion	details about features and probability distribution	2 breast imaging attending radiologists with 18 and 6 years experience and 4 breast imaging fellows radiologists	10 practice cases	NA
Sohns	2010	classification (BI-RADS) and classification (ACR types breast tissue)	marking of suspicious lesions	NA	1 attending physician of unknown specialty and experience and 1 resident physician of unknown specialty and experience	NA	NA
Steiner	2018	classification (negative, isolated tumour cells cluster, micrometastasis, macrometastasis)	heatmap highlighting suspicious regions of interest	NA	6 pathologists with 1-15 years experience	participation in pilot study and 5 practice cases	NA
Stoffel	2018	score assignment (confidence in diagnosis)	system "rating"	NA	1 board certified radiologist with 8 years experience and 1 board certified radiologist with 3 years experience and 1 board certified radiologist with 2 years experience and 1 3rd year radiology resident	NA	NA
Sun	2014	identification (thrombus)	Highlighting of suspicious regions and likelihood score for the presence of a thrombus	NA	3 "senior" radiologists and 2 "junior" radiologists	NA	NA
Sunwoo	2017	identification (metastasis candidates) and score assignment (confidence)	highlighting of suspicious regions and probability score	NA	2 board certified neuroradiologists with 7 years experience and 2 radiology residents with 4 and 2 years experience	NA	NA
Tang	2011	score assignment (confidence in the presence of abnormality)	highlighting of suspicious regions	NA	2 specialised radiologists with 9.5 years experience on average and 2 radiology residents with 6 years experience on average and 2 emergency physicians with 2.5 years experience on average	NA	NA
Taylor	2018	score assignment (confidence in normal findings)	5-points scale (probability of belonging to the disease class)	NA	2 radiologists with more than 5 years experience	NA	Interviews on human-CAD relationship and CAD effects on decision making

© 2021 Vasey B et al. *JAMA Network Open.*

Vassallo	2018	identification (lung metastasis)	highlighting of suspicious regions and nodule measurements	NA	3 radiologists with 3-35 years experience	NA	NA
Wanatabe	2019	classification (normal, recallable)	highlighting of suspicious regions and malignancy probability score	NA	3 mammography fellowship trained radiologists with 5-19 years experience and 4 general radiologists with 1-42 years experience	NA	NA
Way	2010	score assignment (likelihood of malignancy) and prescription (recommended management) and characterisation (features description)	0-10 scale (likelihood of malignancy) and class distribution curves	displays the fitted class distribution	6 fellowship-trained thoracic radiologists with 1-8 years post fellowship experience	one training session	NA
Zhang	2016	score assignment (estimated likelihood of malignancy) and prescription (recom. management)	likelihood of malignancy and 10 features distribution in context of the training set	gives details about features in context of the training set	5 expert radiologists with 12-21 years experience in sonography and 5 radiology residents with "limited experience"	30 practice cases	NA

*commercial name, NA = not available, BI-RADS = breast imaging-reporting and data system, ACL = anterior cruciate ligament, PE = pulmonary embolism, DMIST = Digital Mammographic Imaging Screening Trial, PI-RADS = Prostate Imaging–Reporting and Data System, ROI = region of interest, ACR = American College of Radiology

© 2021 Vasey B et al. *JAMA Network Open.*
